# Molecular phylogeny and systematics of native North American lumbricid earthworms (Clitellata: Megadrili)

**DOI:** 10.1371/journal.pone.0181504

**Published:** 2017-08-09

**Authors:** Csaba Csuzdi, Chih-Han Chang, Tomás Pavlícek, Tímea Szederjesi, David Esopi, Katalin Szlávecz

**Affiliations:** 1 Department of Zoology, Eszterházy Károly University, Eger, Hungary; 2 Department of Earth and Planetary Sciences, Johns Hopkins University, Baltimore, MD, United States of America; 3 Institute of Evolution, University of Haifa, Haifa, Israel; 4 Department of Zoology and Animal Ecology, Plant Protection Institute, Centre for Agricultural Research, Hungarian Academy of Sciences, Budapest, Hungary; 5 Sidney Kimmel Comprehensive Cancer Center, Johns Hopkins University, Baltimore, MD, United States of America; Laboratoire de Biologie du Développement de Villefranche-sur-Mer, FRANCE

## Abstract

The family Lumbricidae is arguably the most well-known and well-studied earthworm group due to its dominance in the European earthworm fauna and its invasion in temperate regions worldwide. However, its North American members, especially the genus *Bimastos* Moore, 1893, are poorly understood. We revised the systematics of the genus *Bimastos* and tested the hypothesis of the monophyly of North American lumbricids using morphological characters and eight molecular markers. Phylogenetic analyses based on our extensive sampling of *Bimastos* and inclusion of *Dendrodrilus* and *Allolobophoridella* indicated a well-supported clade containing *Bimastos* and *Eisenoides* Gates, 1969, and provided the first evidence supporting that North American lumbricids are monophyletic. Assuming the available divergence time estimations and dating of land bridges are correct, it would suggest that the ancestor of this clade arrived North America through Beringia or the De Geer route during Late Cretaceous, and since then the clade has diverged from its Eurasian sister group, *Eisenia*. The peregrine genera *Dendrodrilus* and *Allolobophoridella* are nested within the *Bimastos* clade; we propose to treat them as junior synonyms of the genus *Bimastos*, and, contradictory to the commonly held belief of being European, they are indeed part of the indigenous North American earthworm fauna. Morphological characters, such as red-violet pigmentation, proclinate U-shaped nephridial bladders and calciferous diverticula in segment 10 further support this placement. The East Mediterranean–Levantine *Spermophorodrilus* Bouché, 1975 and *Healyella* Omodeo & Rota, 1989 are nested within the *Dendrobaena sensu lato* clade; therefore their close relationship with the North American *Bimastos* is refuted. Species fit the revised diagnosis of *Bimastos* are reviewed and keyed, and a new species, *Bimastos schwerti* sp. nov., is described.

## Introduction

Earthworms of the family Lumbricidae are native to the Holarctic. They represent a keystone group of macrofauna in temperate soils, with about 30 common species spread globally by human activity [1]. The family currently has around 750 described species within approximately 40–60 genera [2–4]. While the majority of the species and genera are native to the Palearctic region, two genera, *Bimastos* Moore, 1895 and *Eisenoides* Gates, 1969, are generally believed to be native to North America [5], with nine and two described species, respectively. In the USA, species belonging to the two genera frequently co-occur with introduced European lumbricids, such as *Lumbricus rubellus*, *Aporrectodea caliginosa*, and *Octolasion cyaneum* [6–8], but usually at lower abundance.

The validity, taxonomic boundary and origin of *Bimastos*, aptly called a “systematic wastebasket” by Gates [9], have been widely debated [5, 10–16]. Taking into account previously overlooked morphological characters, including the shape and orientation of nephridial bladders and the structure and position of calciferous glands, Gates [5] argued that the name *Bimastos* should be restricted only for North American species. This concept of *Bimastos* was later supported by Omodeo and Rota [14], who separated the Balkanic and Anatolian *Spermophorodrilus* Bouché, 1975 and *Healyella* Omodeo & Rota 1989 from North American *Bimastos*. However, Omodeo and Rota’s concepts of the three genera suffered severely from ambiguous morphological descriptions and overlapping diagnosis. For this reason, Zicsi [13, 15] concluded that the three genera form a homogenous group, and both *Spermophorodrilus* and *Healyella* are junior synonyms of *Bimastos*. In contrast to Gates’ restricted definition of *Bimastos* to the Nearctic, Zicsi’s concept of *Bimastos* encompasses species not only from North America but also from the Balkans and the Anatolia, thus creating a biogeographic puzzle with questions on how this genus achieved its current native range of distribution.

The biogeographic puzzle concerning the two competing hypotheses of *Bimastos* is further complicated by the close affinity among *Bimastos* and two monotypic genera, *Allolobophoridella* and *Dendrodrilus* [17, 18]. This affinity has been suggested in preliminary molecular analyses [19, 20], and was recently confirmed in the multigene molecular phylogeny of Lumbricidae [21]. The genus *Allolobophoridella* was created to host two enigmatic species, *Lumbricus eiseni* Levinsen, 1884 and *Allolobophora parva* Eisen, 1874 [17]. The latter was soon transferred to *Bimastos* [3], making *Allolobophoridella* monotypic. In the past few decades, *Allolobophoridella eiseni* has been moved around among *Allolobophora* [22], *Eisenia* [15], and *Bimastos* [18] and has also been suggested to show affinities with several *Dendrobaena* species [23]. Clearly, a phylogenetic re-evaluation of the species and the status of the genus *Allolobophoridella* were urgently needed.

While morphological similarities, such as proclinate U-shaped nephridial bladders, and Nearctic distributions imply that *Bimastos* and *Eisenoides* may be closely related, a hypothesized North American clade composed of only *Bimastos* and *Eisenoides* were put through a formal test only recently. Molecular phylogeny of Lumbricidae constructed by Dominguez et al. [21] showed that the North American *Bimastos* is monophyletic, and is nested within a clade consisting *Bimastos*, *Dendrodrilus*, and *Allolobophoridella*, with the former two genera being the sister groups of each other. While the hypothesis that *Eisenoides* is closely related to the aforementioned clade gained some support in Dominguez et al. [21], the inferred phylogeny did not support a strict *Bimastos*-*Eisenoides* monophyly. Moreover, the result was insufficient for making definitive inferences regarding the status of *Bimastos*, *Healyella*, and *Spermophorodrilus* as only three out of the nine nominal species of *Bimastos* were included, and the genus *Spermophorodrilus* and the type species of *Healyella* were missing from the analyses. Nevertheless, the molecular phylogenetic study of Lumbricidae by Dominguez et al. [21] provided a solid basis for further analysis.

Here we report on a detailed morphological and molecular analysis of *Bimastos* and related genera. Our objectives were to (1) evaluate the monophyly of North American lumbricids, (2) test the two competing hypotheses of *Bimastos* outlined by Gates (1969) and by Zicsi (1981), (3) investigate the phylogenetic position of the genera *Allolobophoridella*, *Dendrodrilus*, *Spermophorodrilus*, and *Healyella*, and (4) conduct a full revision of the genus *Bimastos*. We integrated morphology with the multigene-phylogeny approach [24–28], using both nuclear and mitochondrial genes to acquire a concatenated sequence length of 5715 bp. We expanded sampling of *Bimastos* from three to eight species and included the type species of all of the concerned genera, including *Allolobophoridella eiseni*, *Bimastos palustris*, *Dendrodrilus rubidus rubidus*, *Eisenoides lonnbergi*, *Healyella syriaca*, and *Spermophorodrilus antiquus*.

## Materials and methods

### Specimens

Between 2000 and 2015 we collected in Turkey, the Levant, Middle East, and in the mid-Atlantic region of North America. We also examined the *Bimastos* collection in the National Museum of Natural History in Washington D.C., where several type specimens are kept, as well as the materials in the Hungarian Natural History Museum where several *Healyella* and *Spermophorodrilus* species are housed. Earthworms were collected by both the diluted formalin method [29] and by digging and hand searching. Specimens were killed in 75% ethanol and fixed in 4% formalin then transferred to 75% ethanol after several days. Specimens used for molecular analysis were preserved in 96% ethanol without formalin fixation. All of the specimens collected and/or examined are permanently archived at either the National Museum of Natural History, Smithsonian Institution (USNM), Washington D.C., USA or the Soil Zoology Collection of the Hungarian Natural History Museum (HNHM), Budapest, Hungary. The detailed catalog numbers of specific specimens sequenced can be found in Table 1. For morphological analysis the following specimens were examined: *Allolobophoridella eiseni* 17 specimens (HNHM/12484, HNHM/14565, HNHM/14743); *Dendrodrilus rubidus* 15 specimens (HNHM/14185, HNHM/14228, HNHM/14445, HNHM/15283, HNHM/16384, HNHM/6519); *Spermophorodrilus antiquus* 10 specimens (HNHM/8857, HNHM/9247, HNHM/15756, HNHM/15819, HNHM/15840); *Healyella syriaca* 16 specimens (HNHM/11131, HNHM/12169, HNHM/14045, HNHM/15141, HNHM/16507); *Healyella jordanis* 14 specimens (HNHM/12914, HNHM/12915, HNHM/12918, HNHM/14620); *Bimastos gieseleri* 11 specimens (USNM 25848); *Bimastos heimburgeri* 44 specimens (USNM 123883, USNM 123879, HNHM/14186, HNHM/14906, HNHM/16498, HNHM/16502); *Bimastos longicinctus* 64 specimens (USNM 24599, USNM 25871, USNM 47867, HNHM/17151); *Bimastos palustris* 34 specimens (HNHM/13039, HNHM/14183, HNHM/14189, HNHM/14215, HNHM/14224, HNHM/14227); *Bimastos parvus* 10 specimens (HNHM/14301, HNHM/14886, HNHM/15170, HNHM/16067, HNHM 16464); *Bimastos schwerti* Holotype (HNHM/16614) Paratypes 39 specimens (HNHM/16615, HNHM/14184, HNHM/4188, HNHM/16500, HNHM/16510, HNHM/16566, HNHM/16567, HNHM/17158); *Bimastos tumidus* 55 specimens (USNM 1164, USNM 123878, USNM 123889, USNM 25848, USNM 19683, HNHM 14193, HNHM/14198,. HNHM/16503, HNHM/16497); *Bimastos welchi* one specimen (USNM 16782); *Bimastos zeteki* eight specimens (USNM 16782, USNM 26214, USNM 123897, USNM 123898).

**Table 1:** Specimens newly collected for phylogenetic analyses and their Hungarian Natural History Museum catalog numbers (HNHM).

Species	HNHM	Locality
*Allolobophoridella eiseni*	15811	Koula Mts., Greece
*Allolobophoridella eiseni*	16448	Col d'Aspin, France
*Aporrectodea caliginosa*	17163	Mayo Beach Park, MD, USA
*Aporrectodea tuberculata*	17164	Mayo Beach Park, MD, USA
*Bimastos heimburgeri*	16498	Gunpowder Falls, USA
*Bimastos heimburgeri*	16502	Smithsonian Environmental Research Center, MD, USA
*Bimastos longicinctus*	17157	Game land 242, Siddonsburg PA, USA
*Bimastos palustris*	16565	Jug Bay Wetlands Sanctuary, MD, USA
*Bimastos parvus*	16357	Wadi Kelt, Israel
*Bimastos schwerti*	16500	Jug Bay Wetlands Sanctuary, MD, USA
*Bimastos schwerti*	16566	Jug Bay Wetlands Sanctuary, MD, USA
*Bimastos schwerti*	17158	Game land 242, Siddonsburg, PA, USA
*Bimastos tumidus*	16497	Gunpowder Falls, USA
*Bimastos tumidus*	16503	Smithsonian Environmental Research Center, MD, USA
*Dendrobaena alpina*	16077	Radjuva Planina, Bulgaria
*Dendrobaena attemsi*	16299	Socolau Valley, Maramures, Romania
*Dendrobaena attemsi*	16468	Palmeira de Faro, Portugal
*Dendrobaena byblica*	16660	Kakopetros, Crete, Greece
*Dendrobaena byblica olympiaca*	15835	Peristeri, Greece
*Dendrobaena octaedra*	16212	Treskovac Mts., Montenegro
*Dendrobaena octaedra*	16528	Payolle Valley, France
*Dendrodrilus rubidus rubidus*	15657	Cerová Highlands, Slovakia
*Dendrodrilus rubidus rubidus*	15816	Istrancha Mts., Turkey
*Dendrodrilus rubidus subrubicundus*	15283	Borşa, Romania
*Eisenia fetida*	17161	Baltimore, MD, USA
*Eisenoides carolinensis*	17160	Hawk Mountain, PA, USA
*Eisenoides lonnbergi*	17159	Plummers Island, MD, USA
*Fitzingeria platyura platyura*	16439	Velem, Hungary
*Healyella jordanis*	16369	Rehaniya, Israel
*Healyella syriaca*	16273	Nahal Tabor, Israel
*Healyella syriaca*	16507	Samandog, Turkey
*Lumbricus rubellus*	17165	Smithsonian Environmental Research Center, MD, USA
*Octolasion lacteum*	17162	Smithsonian Environmental Research Center, MD, USA
*Spermophorodrilus antiquus*	15756	Sapka Mts., Greece

### Ethics statement

Permission to collect earthworm samples at Jug Bay was issued by the Jug Bay Wetlands Sanctuary under Director Chris Swarth. None of the other locations from which samples were collected required specific permissions. None of the earthworms collected in this study are listed as endangered or protected. All of the specimens included in this study are archived in the institutions stated above and are publicly accessible.

### Histological methods

For histological study of the longitudinal musculature, several postclitellar segments were embedded in paraffin, sliced to 10 μm thin cross-sections using a Microm rotary-microtome, and stained with hematoxylin and eosin [30]. For comparison of the structure of calciferous glands, several longitudinal sections of the preclitellar regions were also sliced and treated as above. The microscopic slides were examined and photographed using a Nikon Eclipse 660 DIC microscope.

### Taxon sampling for phylogenetic analysis

To unravel the phylogenetic relationships among *Bimastos*, *Healyella*, *Spermophorodrilus*, *Dendrodrilus* and *Allolobophoridella*, and to test the hypothesis that North American lumbricids are monophyletic, we sampled a total of 14 taxa from the above genera, including two *Bimastos* species reported in Domínguez et al. [21], and both of the known species belonging to the North American native genus *Eisenoides* (Table 1). Our overall taxon sampling comprises eight of the 10 valid species of *Bimastos*, including a new species described in the present study, and fully represents the monotypic genera *Allolobophoridella* and *Dendrodrilus*. We also included nine species (three from GenBank; S1 Table) from *Dendrobaena* and *Fitzingeria* as the two genera are closely related and species in *Healyella* and *Spermophorodrilus* have been classified into *Dendrobaena* [11]. While our sampling of the latter four genera was far from exhaustive, *Healyella*, *Spermophorodrilus* and *Fitzingeria* have only 10, three, and three valid species, respectively [4]. Furthermore, our samples encompassed the type species of all of the targeted genera (*Allolobophoridella eiseni*, *Bimastos palustris*, *Dendrobaena octaedra*, *Dendrodrilus rubidus rubidus*, *Eisenoides lonnbergi*, *Fitzingeria platyura platyura*, *Healyella syriaca*, and *Spermophorodrilus antiquus*, respectively), providing a strong taxonomic basis for drawing unequivocal conclusions. Samples of the lumbricid species *Eisenia fetida*, *Lumbricus rubellus*, *Octolasion lacteum*, *Aporrectodea caliginosa* and *Aporrectodea tuberculata* were also included. Overall, sequences from 34 specimens representing 25 species/subspecies were newly acquired, and were combined with selected taxa from the Lumbricidae dataset reported in Domínguez et al. [21] and Pérez-Losada et al. [31] for phylogenetic analyses.

### DNA extraction, polymerase chain reactions, and sequencing

Genomic DNA was extracted from earthworm tissues using the Qiagen DNeasy Blood and Tissue Kit (QIAGEN, Valencia, CA, USA). Regions of eight molecular markers, including three nuclear rRNAs (18S, 5.8S and 28S), two mitochondrial rRNAs (12S and 16S), and three mitochondrial protein coding genes (cytochrome c oxidase subunit 1 and 2 (COI, COII) and NADH dehydrogenase subunit 1 (ND1)), were acquired using polymerase chain reaction (PCR) with primers listed in S2 Table. PCR was conducted in a 50 ul total volume with 1x reaction buffer, 1.25 units JumpStart Taq Polymerase (Sigma, St Louis, MO, USA), 200 uM of each dNTP, 1.5 mM MgCl_2_, 0.24 mg/mL BSA, 5% DMSO, 200 nM of each primer, and 10 or 20 ng template DNA. Cycling conditions were set to one cycle of 94°C for 2 min, followed by 35 cycles of 94°C for 15 s, 45°C (for COI), 47°C (for COII and ND1), 49°C (for 16S rRNA) or 50°C (for 12S, 18S, 5.8S and 28S rRNAs) for 15 s, and 72°C for 90 s, with a final cycle of 72°C for 5 min. The amplified products were sequenced at Beckman Coulter Genomics using BigDye Terminator v3.1 Cycle Sequencing Kit (Applied Biosystems, Foster, CA, USA) and analyzed on an ABI PRISM 3730XL (Applied Biosystems). Chromatograms were visualized and assembled in DNA Baser v4.31.0 (Heracle BioSoft, Romania). All new sequences have been deposited in GenBank under the accession numbers KX651115-KX651415.

### Phylogenetic analysis

Two datasets were analyzed. First, sequences from selected species representing the major clades of Lumbricidae and the outgroup *Hormogaster* reported in Domínguez et al. [21] and Pérez-Losada et al. [31] were combined with our data (S1 Table). The combined dataset, composed of 12S, 16S, 18S rRNAs, COI, COII, and ND1, contains 64 samples representing 57 species/subspecies and is 3940 bp after alignment (the ‘short dataset’ hereafter). Second, data acquired in this study were analyzed. *Octolasion lacteum* was used as the outgroup based on the inferred phylogeny in Domínguez et al. [21]. This dataset, 5715 bp after alignment, is longer (due to both longer sequences and some longer alignments) and allows us to focus on unravelling the phylogeny within *Bimastos* (the ‘long dataset’ hereafter).

Nucleotide sequences from each gene were aligned using MAFFT v7 [32] under the default settings. The aligned sequences were concatenated using DAMBE 5 [33]. For the short dataset, the aligned sequences are 3940 bp in length, including 18S rRNA (768 bp), 12S rRNA (392 bp), 16S rRNA (516 bp), COI (651 bp), COII (681 bp), and ND1 (932 bp). For the long dataset, the aligned sequences are 5715 bp in length, including 18S rRNA (1578 bp), 5.8 S rRNA (122 bp), 28S rRNA (867 bp), 12S rRNA (388 bp), 16S rRNA (496 bp), COI (651 bp), COII (681 bp), and ND1 (932 bp). The most appropriate models of evolution were selected using jModelTest 2 [34] under the Akaike information criterion (AIC) for each gene partition in each dataset. For each protein-coding gene, the third codon was further treated as a separate partition. Different partitions were treated as unlinked and model parameters were estimated independently for each partition in all analyses.

Phylogenies were inferred using maximum likelihood (ML) analyses and Bayesian inferences. ML analyses were conducted using RAxML v8 [35] as implemented in the CIPRES Science Gateway 3.3 web portal [36](www.phylo.org) using the general time reversible model with proportion of invariable sites and gamma distribution (GTR + I + G) estimated for each individual gene partition. Clade support was evaluated using the non-parametric bootstrapping procedure with 1000 bootstrapping replicates. The best ML tree was compared to alternative tree topologies using the Shimodaira–Hasegawa (SH) test as implemented in RAxML v8. Bayesian inferences coupled with Marko chain Monte Carlo (MCMC) were conducted using MrBayes v3.2.6 [37] with default priors and random starting trees. Three independent MCMC searches, each with three heated and one cold chains, were run for 2 x 10^7^ generations. The resulting trees were sampled every 1000 generations after discarding the first 20% trees as burn-in. The posterior probabilities and the topologies of the resulting consensus trees from separate analyses were compared for congruency and combined in a 50% majority-rule consensus tree.

We originally considered estimating divergence time and conducting ancestral area reconstruction but decided not to do so for three reasons. First, there are no earthworm fossils available for calibration. Molecular clock estimation in earthworms has been conducted exclusively using geological events [21, 24, 26]. However, doing so would imply vicariance, an assumption that has been repeatedly questioned [28]. Second, external calibration points are not available in our phylogenetic trees. Third, with the lack of native lumbricid samples from East Asia and *a priori* knowledge about the origin of *Allolobophoridellaa eiseni* and *Dendrodrilus rubidus*, ancestral area reconstruction cannot be properly conducted.

### Nomenclatural acts

The electronic edition of this article conforms to the requirements of the amended International Code of Zoological Nomenclature, and hence the new names contained herein are available under that Code from the electronic edition of this article. This published work and the nomenclatural acts it contains have been registered in ZooBank, the online registration system for the ICZN. The ZooBank LSIDs (Life Science Identifiers) can be resolved and the associated information viewed through any standard web browser by appending the LSID to the prefix "http://zoobank.org/". The LSID for this publication is: **urn:lsid:zoobank.org:pub:236DD001-3F6B-4D1C-AAB2-D3E08FFEC0B3.** The electronic edition of this work was published in a journal with an ISSN, and has been archived and is available from the following digital repositories: **PubMed Central, LOCKSS**.

## Results

### Phylogeny

In both of the short (3940 bp) and the long (5715 bp) datasets, the maximum likelihood (ML) analyses and Bayesian inferences generate similar topologies and have no incongruence regarding supported clades (bootstrap support values ≥ 50 or posterior probabilities ≥ 0.90). Therefore, the ML and Bayesian trees are considered together and only the ML trees are shown.

**Fig 1:**
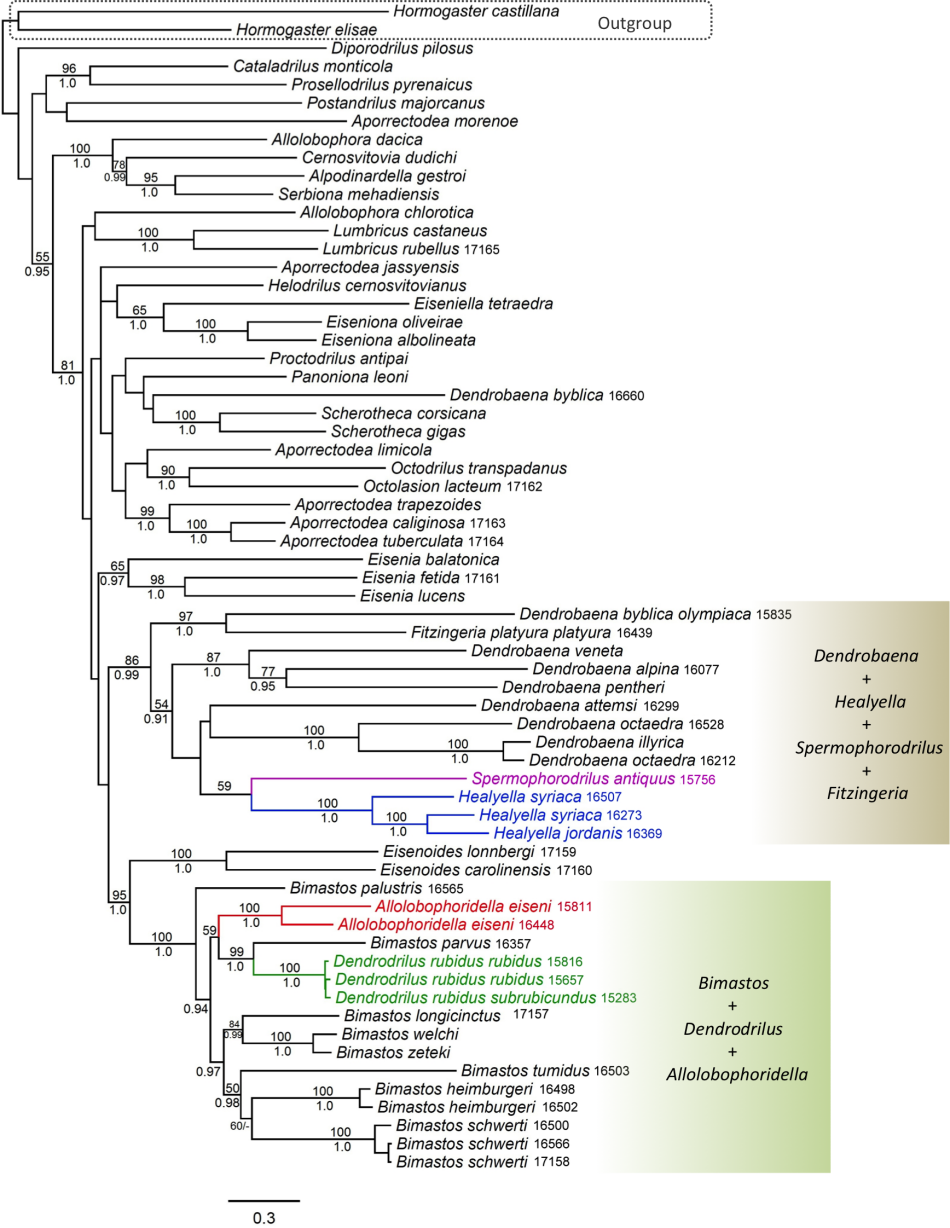
Maximum likelihood tree based on the short dataset for *Bimastos*, *Dendrodrilus*, *Allolobophoridella*, *Healyella*, *Spermophorodrilus*, *Dendrobaena*, and other lumbricids. Bootstrap support values (if ≥ 50) and Bayesian posterior probabilities (if ≥ 0.90) are shown above and below the branches, respectively. The four genera that were previously hypothesized to be related to *Bimastos* are colored. The annotated-and-shaded areas on the right correspond to the two major clades characterized in Results. Specimens newly reported in this study were marked with their five-digit HNHM catalog numbers.

The trees inferred from the short dataset (Fig 1) provided little support for some of the basal internal branches, which is what we expected as the genes included in the short dataset are only a part of those used in Domínguez et al. [21]. As our goal is to understand the phylogenies of *Bimastos*, *Allolobophoridella*, *Dendrodrilus*, *Spermophorodrilus* and *Healyella* with an extended sampling, the lack of phylogenetic resolution among other lumbricid genera does not affect our ability to draw meaningful conclusions, and can reasonably be compensated with our current understanding on Lumbricidae phylogeny [21, 31].

**Fig 2:**
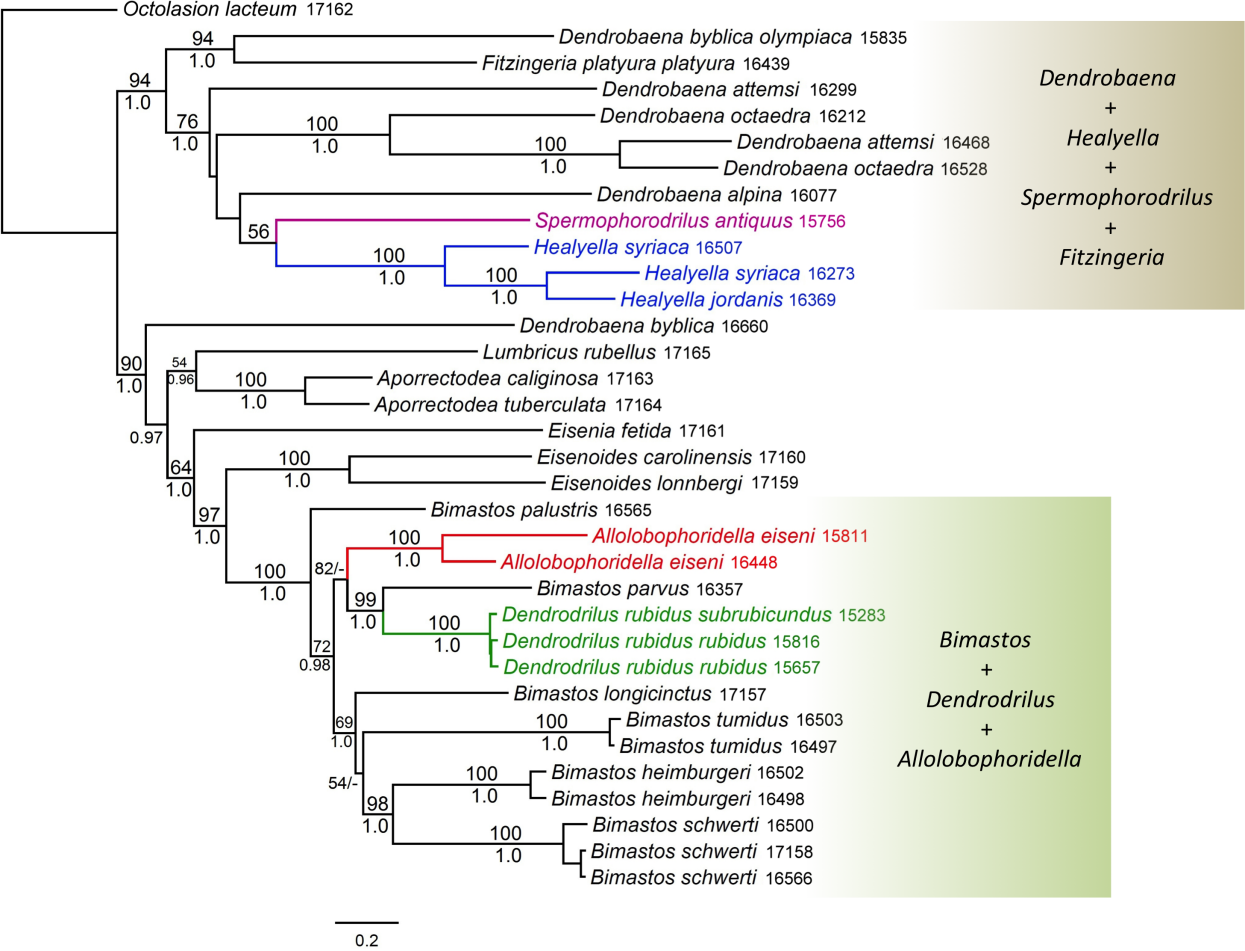
Maximum likelihood tree based on the long dataset for *Bimastos*, *Dendrodrilus*, *Allolobophoridella*, *Healyella*, *Spermophorodrilus*, *Dendrobaena*, and other lumbricids. Bootstrap support values (if ≥ 50) and Bayesian posterior probabilities (if ≥ 0.90) are shown above and below the branches, respectively. The four genera that were previously hypothesized to be related to *Bimastos* are colored. The annotated-and-shaded areas on the right correspond to the two major clades characterized in Results. Specimens were marked with their five-digit HNHM catalog numbers.

The phylogenies inferred from both datasets strongly support a monophyletic group (the *Bimastos* clade hereafter) composed of *Bimastos*, *Allolobophoridella*, and *Dendrodrilus* (bootstrap/posterior probability values = 100/1.0 for both datasets). However, the genus *Bimastos* sensu Gates [5], is paraphyletic due to the exclusion of *Allolobophoridella* or *Dendrodrilus*. Within the *Bimastos* clade, *Bimastos palustris* is basal relative to all the other species. *Bimastos parvus* and *Dendrodrilus rubidus* are sister species; the two together are the sister group of *Allolobophoridella eiseni* (Fig 2). We compared the best ML tree (Fig 2) with two alternative topologies using the SH test with the following constraints: (1) monophyly of the *Bimastos* genus; and (2) monophyly of the *Bimastos* genus except *B*. *parvus*. Both comparisons suggested that the best ML tree and the two alternative topologies are not significantly different from each other (*P* > 0.05).

The sister group of the *Bimastos* clade is *Eisenoides* (bootstrap/posterior probability values = 95/1.0 and 97/1.0 for the short and long datasets, respectively). Together, the two genera form a clade that includes all lumbricid species of North American origin (the North American clade hereafter) (Figs 1 and 2). The phylogenetic trees inferred from the long dataset also suggested that *Eisenia* is the sister group of the North American clade (bootstrap/posterior probability values = 64/1.0).

The tree topologies do not support the hypothesis that *Spermophorodrilus* and *Healyella* are associated with *Bimastos*. Instead, the two genera form a weakly supported clade (bootstrap/posterior probability values = 59/0.85 and 56/0.72 for the short and long datasets, respectively) nested within a clade (bootstrap/posterior probability values = 86/0.99 and 94/1.0 for the short and long datasets, respectively) that also includes *Fitzingeria platyura platyura* and nine *Dendrobaena* taxa. These nine taxa encompass all *Dendrobaena* species included in our analyses except *Dendrobaena byblica byblica*. Accordingly, *Dendrobaena*, as currently defined, is polyphyletic.

### Taxonomic treatment

One of our main research goals was to revise the systematics of the genus *Bimastos*. The inferred phylogenies and the non-significant SH test result suggest two possible relationships among *Bimastos*, *Dendrodrilus* and *Allolobophoridella*: (1) *Bimastos* is paraphyletic due to the existence of *Dendrodrilus rubidus* and *Allolobophoridella eiseni*, or (2) *Bimastos* is monophyletic. Regardless of the relationship, the three groups formed a highly supported clade (Figs 1 and 2). Given the weak morphological distinction among these three genera [38], we prefer an unambiguously supported, more inclusive *Bimastos*, and herein propose to treat *Dendrodrilus* and *Allolobophoridella* as junior synonyms of *Bimastos*. This taxonomic treatment accommodated both scenarios of phylogenetic relationships. Furthermore, Mršič [38], in his original description of *Allolobophoridella*, noted: "Should it be stated, that in the species of the genus *Bimastos* from North America the glandular part of the nephridial bladder is oriented in the same way as in *eiseni* and *parvus*, the genus *Allolobophoridella* will be just a synonym of the genus *Bimastos*."

#### Genus *Spermophorodrilus* Bouché, 1975

*Eophila* Rosa, 1893 [39]: Černosvitov 1938 [40]: 198 (partim).

*Bimastos* Moore, 1893 [41]: Zicsi 1981 [13]: 432 (partim); Zicsi & Michalis 1981 [42]: 244 (partim); Blakemore 2008b [43]: 536 (partim).

*Spermophorodrilus* Bouché, 1975 [44]: 2; Omodeo & Rota, 1989 [14]: 169; 1991[45]: 172; Csuzdi et al. 2006 [46]: 26; Pavlíček et al. 2010 [47]: 2000.

**Fig 3:**
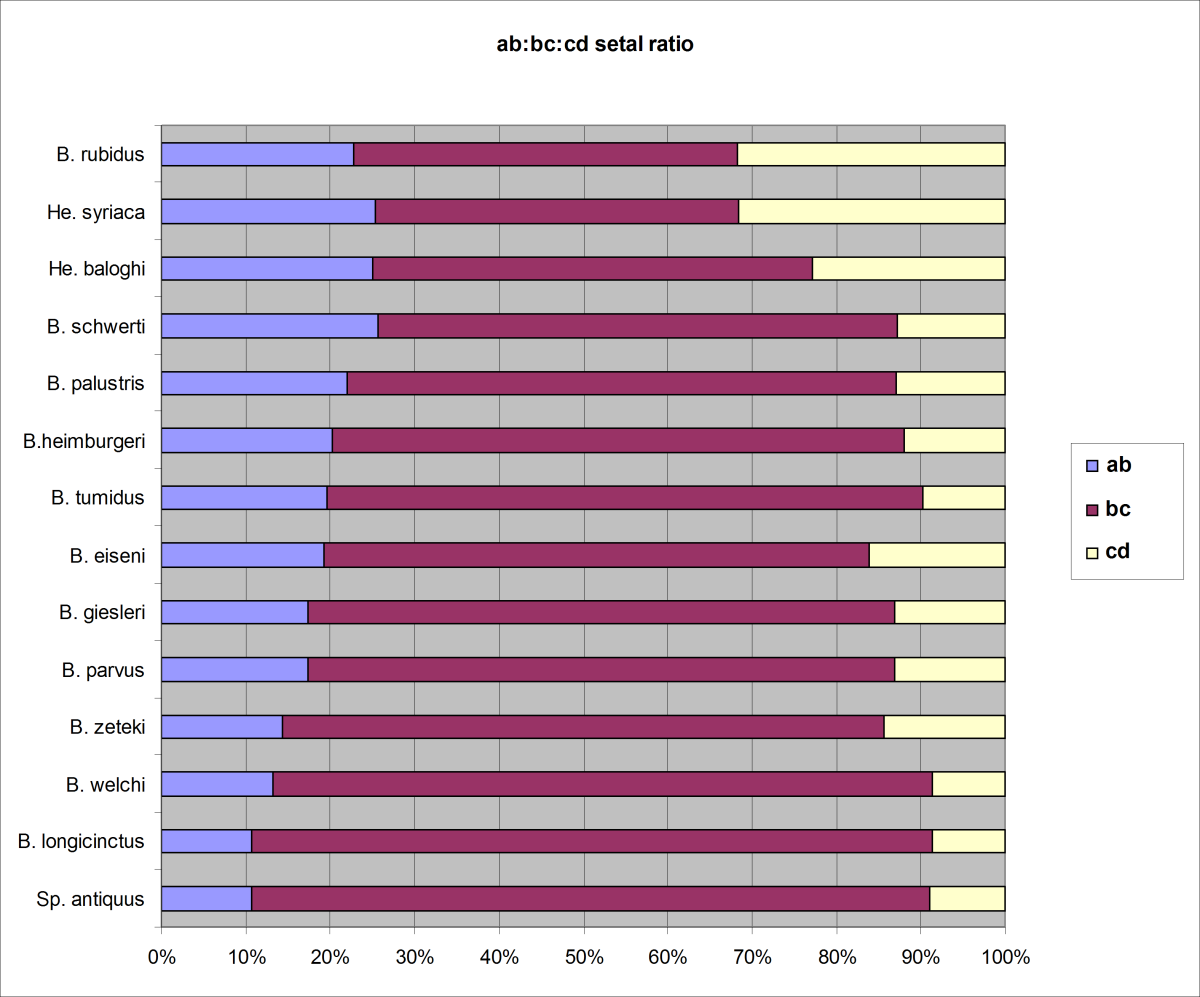
Setal ratios in the genus *Bimastos*. Letters ab, bc, cd refers to setal intervals.

Diagnosis. Setae strictly paired, pigmentation lacking (Fig 3). Prostomium epilobous, first dorsal pore around 5/6. Male pore on 15 large, just above setal line *b*, facing ventrad. Female pores small on 14 just above setae *b*. Clitellum annular, evenly developed, spermathecae and tubercles lacking. Nephridial pores irregularly alternate between *b* and above *d*. Two pairs of testes in 10, 11, and two pairs of seminal vesicles in 11, 12. Calciferous glands in segments 10–12, with small diverticula in segment 10. Excretory system holoic, nephridial bladders sausage-shaped throughout. Typhlosole bifid, the cross-section of longitudinal muscle layer is of pinnate type.

Type species: *Eophila antiqua* Černosvitov, 1938 (= *Spermophorodrilus albanianus* Bouché, 1975)

Distribution. From the Balkan Peninsula to North Anatolia.

Remarks. Omodeo & Rota [14] noted that the species of *Spermophorodrilus* differed from the type species of the genus *Bimastos*, *B*. *palustris*, “*in many relevant points*” (p. 169). However, they did not discuss it in details, and the only difference mentioned is the one segment longer gizzard (17−19 vs. 17−18, respectively). Perhaps the presence of red pigment in *Bimastos* was a key point, but in the Omodeo & Rota’s diagnosis of *Spermophorodrilus*, they stated: “*epidermis devoid or almost devoid of pigment*”, a statement that can reasonably be applied also to *B*. *palustris*, as the live specimen of *B*. *palustris* is almost devoid of pigment throughout its body except for the head dorsad.

#### Genus *Healyella* Omodeo & Rota, 1989

*Helayella* Omodeo & Rota, 1989 [14]: 172; 1991 [45]: 173; Csuzdi et al. 2006 [46]: 20; Blakemore 2008b [43]: 537; Pavlíček et al. 2010 [47]: 1999.

*Bimastos*: Zicsi & Michalis 1993 [15]: 303 (partim); Csuzdi & Pavlíček 1999 [48]: 470; 2002 [49]: 109.

**Fig 4:**
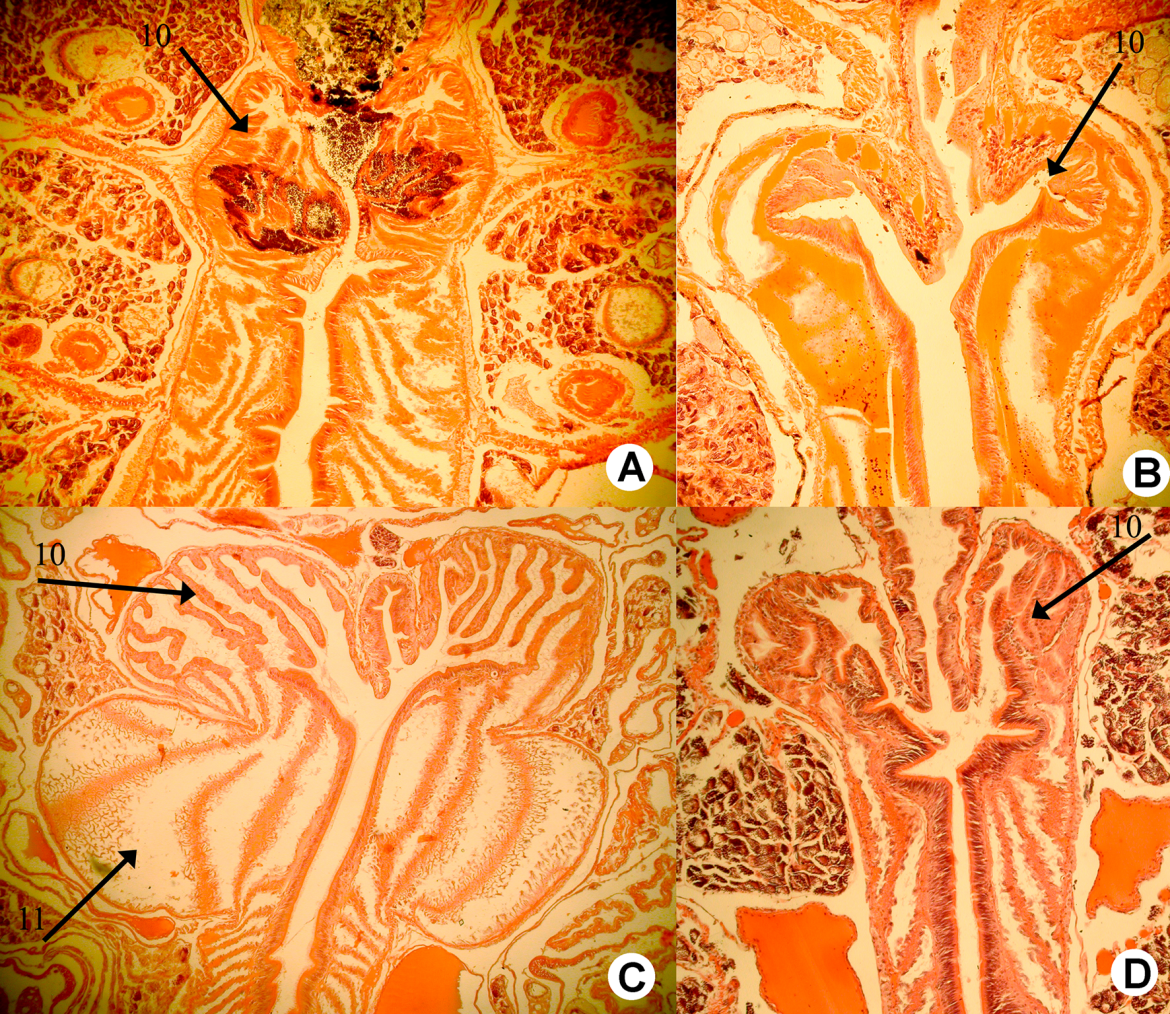
Calciferoous glands in the genera *Bimastos* and *Healyella* (longitudinal sections). A = *Bimastos schwerti* sp. nov., B = *Healyella syriaca* (Rosa, 1893), C = *Bimastos eiseni* (Levinsen, 1884), C = *Bimastos rubidus* (Savign, 1826). Numbers 10 and 11 refer to segments. Arrows point to diverticula.

Diagnosis. Setae moderately paired or distant, pigmentation brownish or purple at least dorsad on the several first segments. Prostomium epilobous, first dorsal pore around 5/6. Male pore on 15 large, facing ventrad between *ab* or just above setal line *b*. Female pores small on 14 between setae *ab* or just above *b*. Clitellum annular, evenly developed, spermathecae and tubercles lacking. Nephridial pores irregularly alternate between *b* and above *d*. Two pairs of testes in 10, 11, and two pairs of seminal vesicles in 11, 12. Calciferous glands in segments 10−12, with moderate diverticula in segment 10 (Fig 4). Excretory system holoic, nephridial bladders sausage-shaped throughout. Typhlosole lamellar or bifid, cross-section of longitudinal muscle layer is of pinnate type.

Type species: *Allolobophora syriaca* Rosa, 1893

Distribution. From West Anatolia to the Levant.

Remarks. Similar to *Spermophorodrilus*, Omodeo and Rota’s concepts of *Healyella* and *Bimastos* suffer overlapping diagnoses. The only distinguishing characters of *Healyella* were the position of the genital pores between setal line *a* and *b* and the calciferous glands devoid of lateral diverticles [14]. However, the description of *Healyella naja* [14] (p. 176) has male pores above *b* and the description of *Healyella schweigeri* [13] (described as *Bimastos schweigeri*) has calciferous diverticula in 10, make the defining characters of *Bimastos* and *Healyella* sensu Omodeo & Rota [14] overlapping. Description of the nephridial bladders, which is the definitive difference [5] between *Bimastos* and the two Eurasian genera, was entirely missing in Omodeo & Rota [14, 45].

#### Genus *Bimastos* Moore, 1893

*Bimastos* Moore, 1893 [41]: 333; Moore 1895 [50]: 473; Gates 1942 [51]: 103 (partim); Gates 1969 [5]: 306; Gates 1975 [52]: 4; Reynolds 1977 [53]: 61; Zicsi 1981 [13] (partim); Gates 1982 [54]: 27; Fender 1985 [55]: 111; Omodeo & Rota 1989 [14]: 169; Mrsic 1991 [17]: 657; Zicsi & Michalis 1993 [15]: 303 (partim); McKey-Fender, Fender & Marshall 1994 [56]: 1338; Qiu & Bouché 1998a [18]: 211 (partim); Blakemore 2008b [43]: 536.

*Bimastus*: Stephenson 1930 [57]: 930; Omodeo 1956 [58]: 178 (partim).

*Allolobophora (Bimastus)*: Michaelsen 1899 [59]: 13.

*Helodrilus (Bimastus)*: Michaelsen 1900 [10]: 501 (partim); Smith 1917 [60]: 169.

*Eisenia*: Pop 1941 [11]: 509 (partim); Zicsi 1982 [61]: 443 (partim).

*Allolobophora (Allolobophoridella)* Mršić, 1990 [38]: 49. **syn. nov.**

*Allolobophoridella*: Mrsic 1991 [17]: 252; Csuzdi & Zicsi 2003 [3]: 69; Blakemore 2008b [43]: 500.

*Dendrobaena (Dendrodrilus)* Omodeo, 1956 [58]: 175. **syn. nov.**

*Dendrodrilus*: Perel 1976 [12]: 834; 1979 [62]: 200; Mršić 1991 [17]: 260, Csuzdi & Zicsi 2003 [3]: 131; Blakemore 2008b [43]: 562.

Diagnosis. Setae strictly or moderately paired, pigmentation red-violet at least dorsad on the several first segments. Prostomium epi- or tanylobous, first dorsal pore around 5/6. Male pore on 15 large, just above setal line *b*. Female pores small on 14 just above setae *b*. Clitellum annular or saddle-shaped. Spermathecae usually lacking, if present, frequently empty in 9/10-10/11 and open in setal line *c*. Tubercles usually lacking, if present, indistinct bands on the ventral edge of the clitellum. Nephridial pores irregularly alternate between *b* and above *d*. Two pairs of testes in 10, 11, and two pairs of seminal vesicles in 11, 12 (sometimes lacking or three pairs in 9, 10, 11). Calciferous glands in segments 10–12, 13 with variable sized diverticula in segment 10 (Fig 4). Excretory system holoic, nephridial bladders in the anterior part of the body U-shaped with proclinate ental limb, that might partly merge with the ectal limb toward the hind end of the body. Typhlosole lamellar or bifid, the cross-section of longitudinal muscle layer is of pinnate or fasciculated type.

Type species: *Bimastos palustris* Moore, 1895.

Distribution. Western Canada (Vancouver Island) and eastern USA. Several species are peregrine, and introduced all over the temperate regions.

Remarks. This extended definition of *Bimastos* includes *Lumbricus eiseni* Levinsen, 1884 (placed previously in *Allolobophoridella* by Mršić [17]) and also *Dendrodrilus rubidus* (Savigny, 1826). It keeps apart, however, the species of *Healyella* and *Spermophorodrilus* differing in the structure of the nephridial bladders, which are simple, sausage-shaped throughout the body in *Healyella* and *Spermophorodrilus* and U-shaped in at least the first several segments in *Bimastos* (Table 2). Although it is clear that *Spermophorodrilus*, *Healyellla* and *Bimastos* are distinct clades based on Domínguez et al. [21] as well as our study, and that there are numerous taxonomic descriptions in the literature [13–15], we have summarized the differences among these clades in Table 2 in order to resolve the long standing taxonomic confusions and help both taxonomists and ecologists better distinguishing them. In the genus *Bimastos* the phoral insemination prevails. It means that the spermathecae disappeared and the sperm of the copulating partner is stored in spermatophores [63, 64]. This type of insemination is regarded as a plesiomorphy in annelids; however in Lumbricidae it is surely a secondary reversion from thecal (by means of spermathecae) insemination [63]. Spermatophores can be of variable shape from the falciform in *B*. *palustris* to the flattened spermatophores found in *B*. *schwerti* sp. n. and usually they are attached to the body in the region of either the male pores or the clitellum. As they are present only for a short period after copulation, the species without spermathecae were frequently regarded as parthenogenetic [65, 66].

**Table 2:** Morphological comparison among the genera *Bimastos*, *Spermophorodrilus* and *Healyella*. Characters distinguishing one genus from the other two are in boldface.

Character	*Bimastos* Moore, 1993	*Spermohorodrilus* Bouché, 1975	*Healyella* Omodeo & Rota, 1989
Prostomium	Epi- or tanylobous	Epilobous	Epilobous
Clitellum	Annular or saddle-shaped	Annular	Annular
Tubercles	Usually absent	Absent	Absent
Genital pores	Above *b*	Within *ab* or above *b*	Within *ab* or above *b*
Setae	Moderately or closely paired	Closely paired	Moderately paired or distant
**Pigmentation**	Red-violet	**Absent**	Red-violet
Spermathecae	Usually absent	Absent	Absent
**Nephridial bladders**	**Proclinate U-shaped**	Sausage-shaped	Sausage-shaped
Longitudinal muscle	Pinnate or fasciculated	Pinnate	Pinnate
Calciferous gland diverticula in 10	Variable	Small	Moderate

#### *Bimastos eiseni* (Levinsen, 1884) comb. nov

Figs 3 and 5

**Fig 5:**
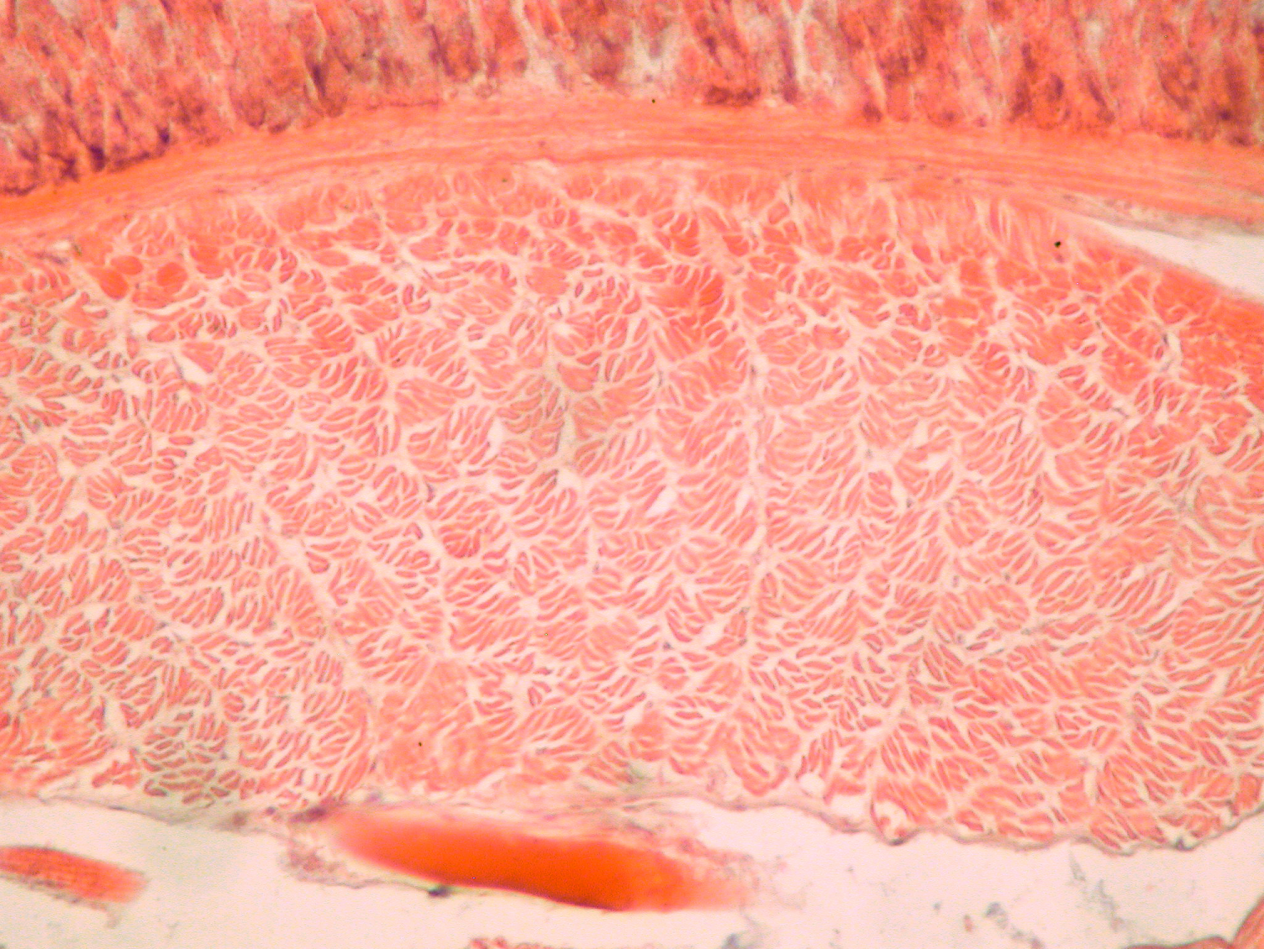
Longitudinal musculature of *B*. *eiseni* (cross section).

*Lumbricus eiseni* Levinsen, 1884 [67]: 241.

*Allolobophora eiseni*: Rosa 1893 [39]: 462; Easton 1983 [68]: 475; Zicsi 1991 [22]: 182.

*Allolobophora* (*Bimastus*) *eiseni*: Michaelsen 1900 [10]: 503.

*Allolobophora rubra* Bretscher, 1900 [69]: 454.

*Dendrobaena merciensis* Friend, 1911 [70]: 192.

*Bimastos eiseni gracilis* Friend, 1911 [70]: 368.

*Bimastus oltenicus* Pop, 1938 [71]: 146.

*Eisenia parva* f. *typica* (part.): Pop 1949 [72]: 89.

*Bimastus eiseni*: Omodeo 1956 [58]: 178.

*Eisenia parva*: Zicsi 1959 [73]: 182.

*Eisenia eiseni*: Zicsi 1968 [74]: 132; 1982: 443.

*Allolobophora* (*Allolobophora*) *eiseni*: Perel 1979 [62]: 187.

*Bimastos eiseni*: Fender 1985 [55]: 110; Qiu & Bouché 1998a [18]: 197.

*Allolobophoridella eiseni*: Mršić, 1991 [17]: 255; Reynolds 1995 [75]: 10; Csuzdi & Zicsi 2003 [3]: 69; 1999 [2]: 999; Blakemore 2008b [43]: 499.

Diagnosis. Body length 30–85 mm, diameter 2–4 mm. Color dark red-violet dorsally and paler ventrally. Prostomium tanylobous, first dorsal pore in 5/6. Setae strictly paired (Fig 3), glandular tumescences usually on 16 *ab*. Clitellum 24, 25–32 saddle-shaped. Male pore on 15, equatorial just above setae *b*, on a porophore bulging somewhat into the neighbouring segments. Female pore on 14 small, dorsad of *b*. Nephropores irregularly alternate between *b* and above *d*. No septa notably thickened. Calciferous glands in segments 10–12 with large diverticula in segments 10, 11 (Fig 4). Excretory system holoic. Nephridial bladders U-shaped throughout, with forward-bent ental limbs. Typhlosole well developed, lamellar. The cross-section of longitudinal muscle layer is of fasciculated type (Fig 5).

Remarks. With no knowledge on the nepheridial system of *B*. *palustris* and the majority of North American *Bimastos*, Mršić [17] kept his *Allolobophoridella* separated from *Bimastos*, but noted that if the nephridial bladders of the North American genus *Bimastos* is “*oriented in the same way as in* eiseni……*the genus* Allolobophoridella *will be just a synonym of* Bimastos” [17]. It turns out Mršić was correct on this point. Pop [11] erroneously synonymised *B*. *eiseni* with *B*. *parvus*, causing a long lasting confusion. Perel [62] demonstrated that the two species names should not be in synonymy because *eiseni* has fasciculated type of musculature, whereas *parvus* has pinnate type of musculature. Furthermore, the prostomium of *B*. *eiseni* is tanylobous and not epilobous as in case of *B*. *parvus*. Our molecular results completely support this view.

#### *Bimastos gieseleri* (Ude, 1895)

Figs 3 and 6

**Fig 6:**
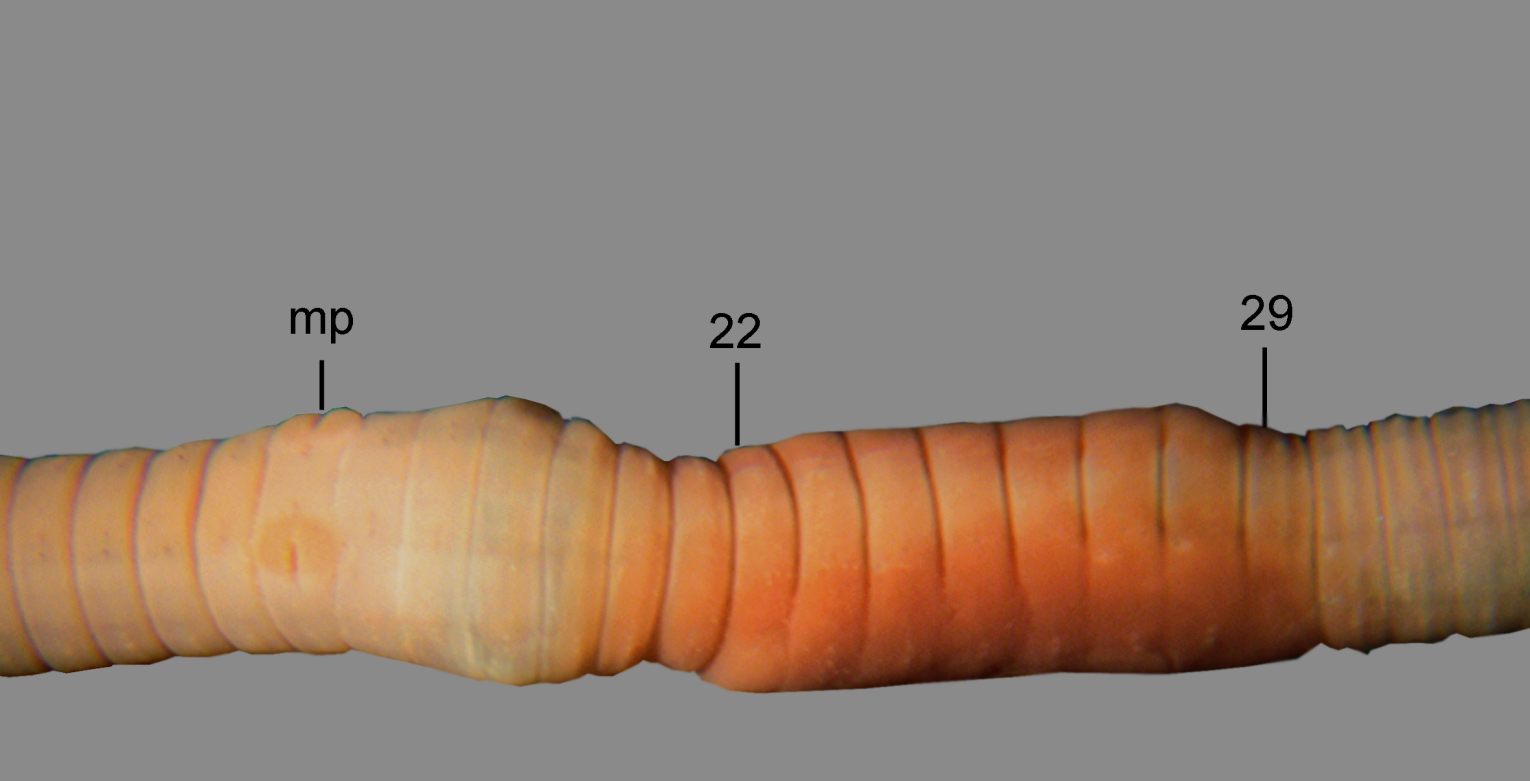
*B*. *giesleri*, ventral view of the clitellar region. mp = male pore, numbers refer to segment numbers.

*Allolobophora gieseleri* Ude, 1895 [76]: 127.

*Allolobophora (Bimastus) gieseleri*: Michaelsen 1899 [59]: 16.

*Helodrilus (Bimastus) gieseleri*: Michaelsen 1900 [10]: 502.

*Helodrilus (Bimastus) gieseleri* forma typica: Smith 1917 [60]: 171.

*Bimastos giessleri*: Gates 1942 [51]: 103; 1982 [54]: 27; Reynolds and Wetzel 2004 [77]: 83; Blakemore 2008b [43]: 538.

*Bimastos giessleri gieseleri*: Blakemore 2008a [78]: 5.

*Bimastos tumidus*: Gates 1956 [65]: 1 (partim); 1969 [5]: 306 (partim); Zicsi 1981 [13]: 433 (partim); Schwert 1990 [79]: 353 (partim).

Material examined. USNM 25848, 11 ex. Florida, USA. 03. 1896. Leg. A. Hempel.

Diagnosis. Body length 65–100 mm, diameter 2–2.5 mm. Color slightly red dorsally and paler ventrally. Prostomium epilobous, first dorsal pore in 5/6. Setae strictly paired (Fig 3), glandular tumescences lacking. Clitellum almost annular but ventrally less developed, on ½21, 22–29, ½30. Male pore on 15, equatorial just above setae *b*, on a porophore bulging somewhat into the neighbouring segments (Fig 6). Female pore on 14 small, dorsad of *b*. Nephropores irregularly alternate between *b* and above *d*. Septa 6/7, 10/11–14/15 slightly, 7/8–9/10 moderately thickened. Calciferous glands in segments 10–12 with diverticula in segment 10. Excretory system holoic. Nephridial bladders U-shaped throughout, with forward-bent ental limbs. Typhlosole well developed, bifid. There is no data on the structure of the longitudinal musculature.

Remarks. *B*. *gieseleri* is similar to *B*. *tumidus*, but differs from it in the presence of thickened septa, the bifid type of the typhlosole and by its larger size. Therefore, in line with other authors (e.g. [43, 78, 79]) we regard it as a valid species.

#### *Bimastos heimburgeri* (Smith, 1928)

Figs 3 and 7

**Fig 7:**
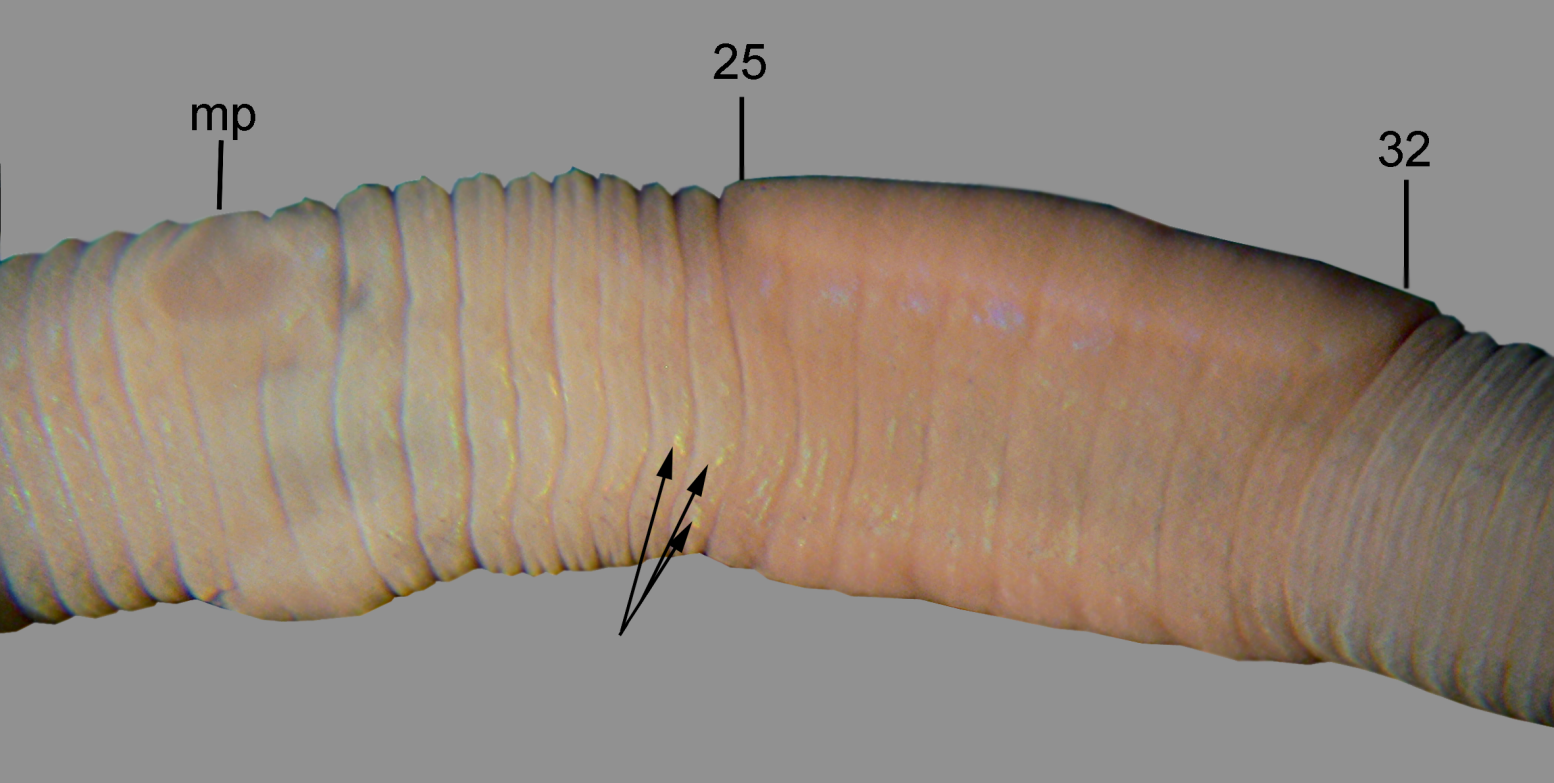
*B*. *heimburgeri*, ventral view of the clitellar region. mp = male pore, numbers refer to segment numbers, arrows point to the spermatophores.

*Helodrilus heimburgeri* Smith, 1928 [80]: 353.

*Bimastos heimburgeri*: Gates 1942 [51]: 103; Gates 1969 [5]: 306; Reynolds et al. 1974 [81]: 25; Zicsi 1981 [13]: 433; Schwert 1990 [79]: 353; Reynolds and Wetzel 2004 [77]: 83; Blakemore 2008a [78]: 5, 2008b [43]: 538.

Material examined. USNM 123883, 15 ex. 4 mi towards Durham, Orange Co., N. Carolina, USA. 29. 02. 1972. Leg. R. Crawford and P. Jinright. USNM 123879, 15 ex. William B. Umstead State Park, Wake County, North Carolina, USA. 11. 01. 1972. Leg. R. Crawford and P. Jinright. New Records: HNHM/14186, 8 ex. On the bank of a streamlet near to the Visitor Center, Jug Bay Wetlands Sanctuary, Anne Arundel Co. MD, USA. 27. 04. 2001. Leg. Cs. Csuzdi and K. Szlávecz. HNHM/14906, 1 ex. Louisville, KY, USA. 22.06.2004. Leg. K. Szlávecz. HNHM/16498, 4 ex. Gunpowder Falls, MD, USA. 19.05.2012. Leg. Cs. Csuzdi and K. Szlávecz. HNHM/16502, 1 ex. Smithsonian Environmental Research Center, Edgewater, MD, USA. 18.05.2012. Leg. Cs. Csuzdi and Ch-H. Chang.

Diagnosis. Body length 40–70 mm, diameter 3–4 mm. Color dark red dorsally and paler ventrally. Prostomium epilobous, first dorsal pore in 5/6. Setae closely paired (Fig 3), glandular tumescences lacking. Clitellum almost annular but ventrally less developed, extends on segments ½24, 25–32, ½33. Male pore on 15, equatorial, just above setae b, on an oval porophore intruding into the neighbouring segments. Female pore on 14 small, slightly dorsad of *b*. Nephropores irregularly alternate between *b* and above *d*. Flat spermatophores present in 20/21, 23, 24 at the setae *ab* (Fig 7). No notably thickened septa. Calciferous glands in segments 10–12 with large diverticula in segment 10. Excretory system holoic. Nephridial bladders U-shaped throughout, with forward-bent ental limbs. Typhlosole bifid. There is no data on the structure of the longitudinal musculature.

Remarks. This species was thought to be parthenogenetic [66, 81]; however, the presence of spermatophores in the specimens examined indicates biparental reproduction with phoral insemination [63, 64].

#### *Bimastos lawrenceae* Fender, 1994

*Bimastos lawranceae* Fender, 1994 in McKey-Fender *et al*. 1994 [56]: 1338; Reynolds & Wetzel 2004 [77]: 83; Marshall & Fender 2007 [82]: 34; Blakemore 2008 [78]: 5.

Diagnosis. Body length and diameter unknown. Color reddish dorsally and paler ventrally. Prostomium epilobous, first dorsal pore in 5/6. Setae closely paired, glandular tumescences lacking. Clitellum saddle-shaped on segments 25–34, 35. Male pore unknown, most likely on 15. Female pore unknown. Nephropores irregularly alternate between *b* and above *d*. Septa 12/13–14/15, moderately thickened. Calciferous glands in segments 10–12 with diverticula in segment 10. Excretory system holoic. Nephridial bladders U-shaped throughout, with forward-bent ental limbs. Typhlosole bifid. There is no data on the structure of the longitudinal musculature.

Remarks. Unfortunately, the original description is incomplete. Most importantly, the descriptions of the male pore and biometry are missing. Fender [56] compared *B*. *lawranceae* with *B*. *zeteki* and wrote that the new species is slightly smaller than *B*. *zeteki*, which is the largest species in the genus with 100–135 mm by 5-6 mm measures. Therefore *B*. *lawranceae* might be around 100 mm long.

#### *Bimastos longicinctus* (Smith and Gittins 1915)

Figs 3 and 8

**Fig 8:**
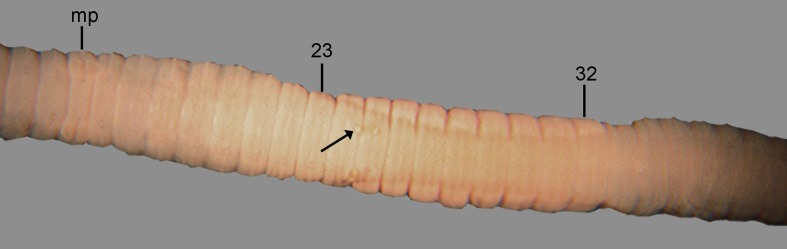
*B*. *longicinctus*, ventral view of the clitellar region. mp = male pore, numbers refer to segment numbers, arrow point to a spermatophore.

*Helodrilus (Bimastus) longicinctus* Smith and Gittins, 1915 [83]: 548; Smith 1917 [60]: 174.

*Bimastos longicinctus*: Gates 1942 [51]: 103; 1969 [5]: 306; Reynolds et al. 1974 [81]: 26; Gates 1982 [54]: 27; Schwert 1990 [79]: 353; Reynolds and Wetzel 2004 [77]: 83; Blakemore 2008a [78]: 5.

*Bimastos parvus*: Gates 1972 [9]: 87, Blakemore 2008b [43]: 538.

Material examined. USNM 24599 *Holotype*. Urbana Illinois. 05. 04. 1911. Leg. F. Smith. USNM 25871 *Paratypes* 12 ex. Urbana, Illinois, USA. 28. 04. 1910. Leg. F. Smith. USNM 47867, 50 ex. Rail Road & Highway Bridge, SW Of Sharon, Route 211, York County, South Carolina, USA. 13. 11. 1962. Leg. W. Murchie. New record: HNHM/17158, 1 ex. Gameland 242, Siddonsburg, PA, USA. 23.04.2013. Leg. Ch-H. Chang, K. Szlávecz and M. Bernard.

Diagnosis. Body length 60–70 mm, diameter 2–3 mm. Color reddish dorsally and paler ventrally. Prostomium epilobous, first dorsal pore in 5/6. Setae closely paired (Fig 3), glandular tumescences lacking. Clitellum saddle-shaped, ventrally extends to setae *a* on segments ½22, 23–32, ½33. Male pore on 15, equatorial just above setae *b*, surrounded by prominent glandular crescents bulging slightly into the neighbouring segments. Female pore on 14 small, dorsad of *b*. Nephropores irregularly alternate between *b* and above *d*. Flat spermatophores are scattered ventrally on the clitellar region (Fig 8). Septa on 6/7 slightly thickened, on 7/8–11/12 strongly thickened. Calciferous glands in segments 10–12 with moderate diverticula in segment 10. Excretory system holoic. Nephridial bladders U-shaped throughout, with forward-bent ental limbs. Typhlosole bifid with manicate limbs. There is no data on the structure of the longitudinal musculature.

Remarks. Based upon the clitellar position, this species is very close to *B*. *heimburgeri*, but differs from it by its paler color, and the presence of thickened septa. The structure of the clitellum is also somewhat different, being almost circular in *B*. *heimburgeri* and ventrally incomplete in *B*. *longicinctus*. Some authors consider *B*. *longicinctus* to be a synonym of *B*. *parvus* (cf. [9, 43]). However, the presence of thickened septa, larger size and longer clitellum clearly separate the two species and, as demonstrated in the present study, they are also genetically distinguishable.

#### *Bimastos palustris* Moore, 1895

Figs 3, 9 and 10

**Fig 9:**
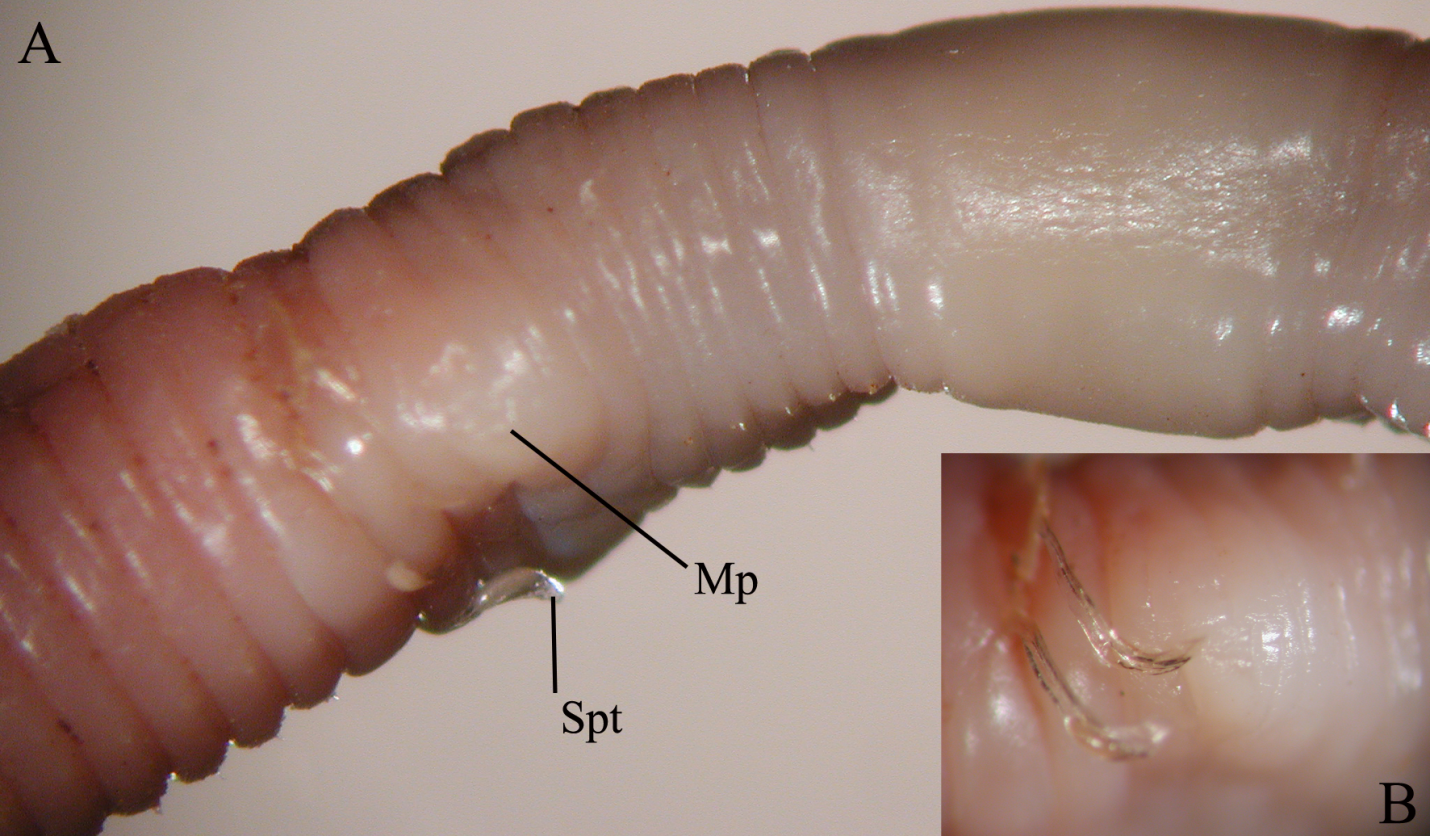
*B*. *palustris*, A = ventrolateral view of the clitellar region, Spt = spermatophore, Mp = male pore. B = Enlarged view of the male pore with falciform spermathophores.

**Fig 10:**
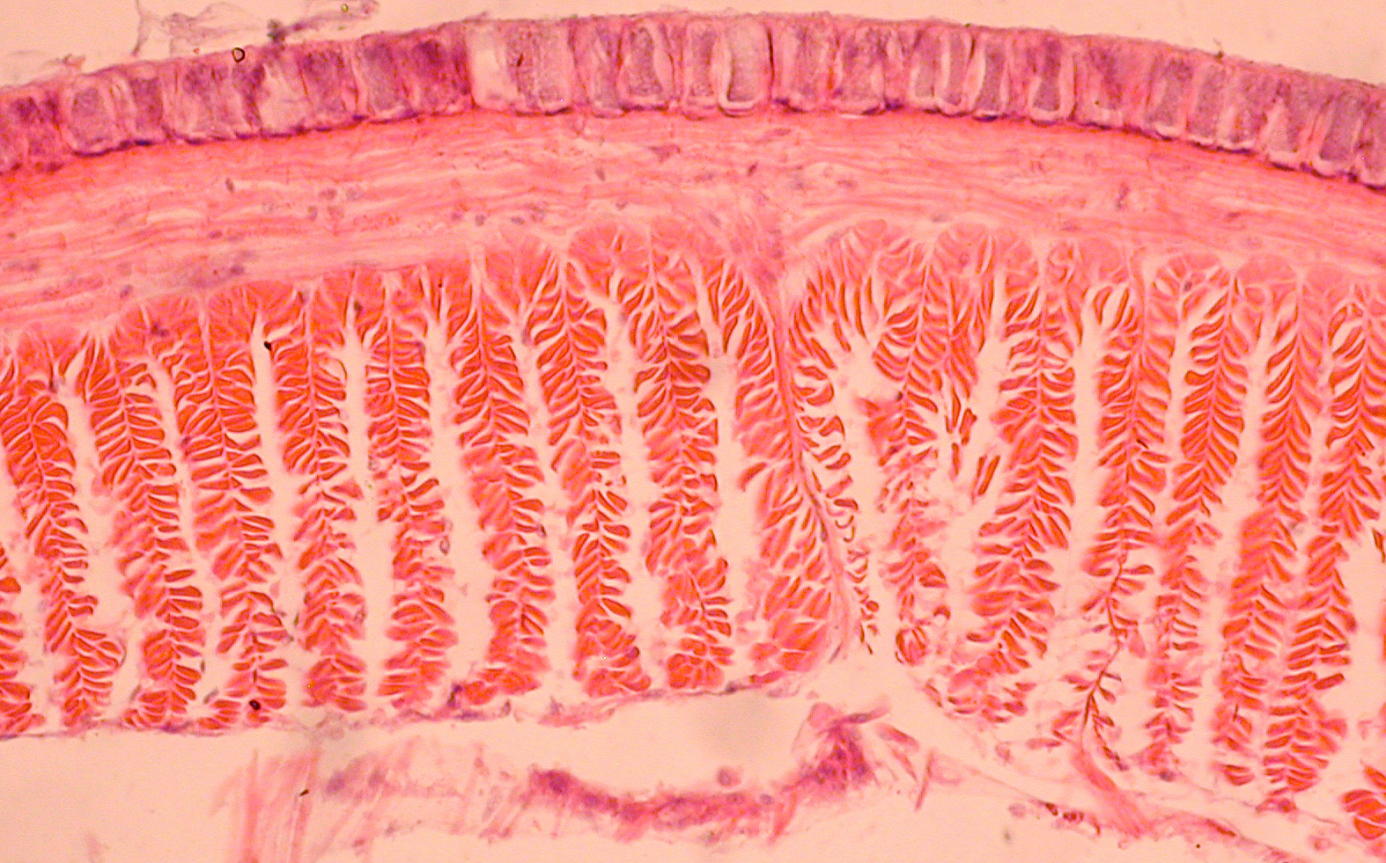
Longitudinal musculature of *B*. *palustris* (cross section).

*Bimastos sp*. Moore, 1893 [41]: 333.

*Bimastos palustris* Moore, 1895 [50]: 473; Gates 1942 [51]:103; 1956 [65]: 9; 1969 [5]: 306; Reynolds *et al*. 1974 [5]: 27; Zicsi 1981 [13]: 433; Gates 1982 [54]: 27; Schwert 1990 [79]: 353; Reynolds and Wetzel 2004 [77]: 83.

*Allolobophora (Bimastus) palustris*: Michaelsen 1899 [59]: 16.

*Helodrilus (Bimastus) palustris*: Michaelsen 1900 [10]: 502; Smith 1917 [60]: 169.

*Bimastus palustris*: Omodeo 1956 [58]: 178; Zicsi 1981 [13]: 433.

*Eisenia palustris*: Zicsi 1982 [61]: 443.

Material examined. New Records: HNHM/13039, 2 ex. Smithsonian Ecological Research Centre, Edgewater, Anne Arundel Co. MD, USA. 18. 04. 1999. Leg. K. Szlávecz, HNHM/14183, 18 ex. On the bank of a streamlet near to the Visitor Center, Jug Bay Wetlands Sanctuary, Anne Arundel Co. MD, USA. 27. 04. 2001. Leg. Cs. Csuzdi and K. Szlávecz, HNHM/14189, 2 ex. Railroad trail, Jug Bay Wetlands Sanctuary, Anne Arundel Co. MD, USA. 27. 04. 2001. Leg. Cs. Csuzdi and K. Szlávecz, HNHM/14215, 5 ex. On a stream bank, Oregon Ridge Nature Park, Baltimore County, MD, USA. 26. 04. 2001. Leg. Cs. Csuzdi and K. Szlávecz, HNHM/14224, 4 ex., 14227, 3 ex., Smithsonian Environmental Research Center, Anne Arundel Co. MD, USA. 28. 04. 2001. Leg. Cs. Csuzdi and K. Szlávecz.

Diagnosis. Body length 18–30 mm, diameter 1.5–2.5 mm. Color pale with reddish hints on dorsum. Prostomium epilobous, first dorsal pore in 5/6. Setae moderately paired (Fig 3), glandular tumescences lacking. Clitellum annular extends on segments 23–28. Male pore on 15, postsetal and ventral, just above setae b, surrounded by well-developed glandular crescents (Fig 9). Female pore on 14 small, slightly dorsad of *b*. Nephropores irregularly alternate between *b* and above *d*. Septa 7/8–8/9 slightly thickened. Crop in 15–16, gizzards small in 17–18. Calciferous glands in segments 10–12 with moderate diverticula in segment 10. Sperm duct open through a muscular copulatory chamber. Excretory system holoic. Nephridial bladders proclinate U-shaped throughout. Typhlosole small, lamellar, the cross-section of longitudinal muscle layer is of pinnate type (Fig 10).

Remarks. The species, contrary to the widely accepted view [66], does not seem to be parthenogenetic but biparental with phoral insemination. Almost all specimens collected in April bore several falciform spermatophores attached around the male pore (Fig 9).

#### *Bimastos parvus* (Eisen, 1874)

*Allolobophora parva* Eisen, 1874 [84]: 46.

*Allolobophora beddardi* Michaelsen, 1894 [85]: 182.

*Allolobophora (Bimastus) parva*: Michaelsen 1899 [59]: 14.

*Allolobophora (Bimastus) beddardi*: Michaelsen 1899 [59]: 13.

*Helodrilus* (*Bimastus*) *parvus*: Michaelsen 1900 [10]: 502; Smith 1917 [60]: 173.

*Helodrilus (Bimastus) beddardi*: Michaelsen 1900 [10]: 502 (partim); Smith 1917 [60]: 173.

*Bimastos beddardi*: Gates 1942 [51]: 103; 1969 [5]: 306; 1982 [54]: 27; Reynolds *et al*. 1974 [81]: 24; Schwert 1990 [79]: 353; Reynolds and Wetzel 2004 [77]: 83.

*Bimastos parvus*: Gates 1942 [51]: 103; 1956 [65]: 6; 1969 [5]: 306; Reynolds *et al*. 1974 [66]: 27; Reynolds 1977 [53]: 61; Gates 1982 [54]: 27; Schwert 1990 [79]: 353; Qiu & Bouché 1998a [18]: 197; Reynolds and Wetzel 2004 [77]: 83; Blakemore 2008a [78]: 5; 2008b [43]: 537.

*Eisenia parva*: Bouché 1972 [86]: 386; Zicsi 1982 [61]: 436.

*Allolobophoridella parva*: Mršić, 1991 [17]: 257.

Non *Bimastos beddardi sophiae* Mercandal de Barrio & Barrio, 1988 [87]: 2 [= *Aporrectodea rosea* (Savigny, 1826) **syn. nov**.]

Material examined. HNHM/14301, 2 ex. Ein Cedem, Mount Carmel, Israel. 10.11.2001. Leg. T. Pavlíček. HNHM/15170, 1 ex. Kibutz Yagur, garden Center, Israel. 11.08.2005. Leg. T. Pavlíček. New Records: HNHM/14886, 4 ex. North Suna, Jordan. 13.03.2005. Leg. T. Pavlíček. HNHM/16067, 2 ex. South Tirol, Austria. 26.10.2011. Leg. T. Peham. HNHM/16464, 1 ex. Rio Cévada, Barcelos, Portugal. 10.09.2012. Leg. T. Pavlíček.

Diagnosis. Body length 20–35 mm, diameter 2–2.5 mm. Color reddish-brown dorsally and paler ventrally. Prostomium epilobous, first dorsal pore in 5/6. Setae closely paired (Fig 3), genital papillae usually lacking. Clitellum saddle-shaped, 24, 25–30, 31, 1/n32. Male pore on 15, equatorial just above setae *b*, on a small porophore confined to its own segment. Female pore on 14 small, dorsad of *b*. Nephropores irregularly alternate between *b* and above *d*. No septa notably thickened. Calciferous glands in segments 10–12 with small diverticula in segment 10. Excretory system holoic. Nephridial bladders U-shaped throughout, with forward-bent ental limbs. Typhlosole well developed lamelliform. The cross-section of longitudinal muscle layer is of pinnate type.

Remarks. *B*. *parvus* was introduced all over the world in the temperate regions. The species was placed in synonymy with other *Bimastos* species multiple times resulting in further confusion within the genus. In Europe, it was frequently lumped together with *Bimastos eiseni* (Levinsen, 1884) because of false synonymyzation by Pop [11, 22, 74]. These European data probably refer to the latter species, because they report on clitellum extending 32, 33. Gates [9] put *B*. *longicinctus* in synonymy of *B*. *parvus* also extending erroneously the clitellar position to 32, 33. *Bimastos beddardi* (Michaelsen, 1894) is usually placed in synonymy of *B*. *parvus* [9, 43, 55, 61, 65]. Because the somewhat longer clitellum (24, 25–31, 1/n32) fits well into the range given for *B*. *parvus* here we support this placement. Mercandal de Barrio & Barrio [87] suggested a new subspecies, *Bimastos beddardi sophiae*. According to the original description and the accompanied figure, this name refers to a parthenogenetic morph of the widely distributed peregrine *Aporrectodea rosea* (Savigny, 1826).

#### *Bimastos rubidus* (Savigny, 1826) comb. nov

Figs 3, 4D and 11

**Fig 11:**
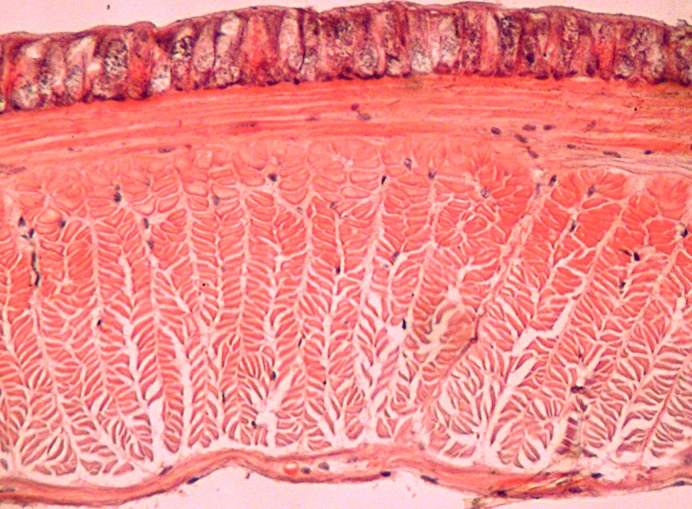
Longitudinal musculature of *B*. *rubidus* (cross section).

*Enterion rubidum* Savigny, 1826 [88]: 182.

*Allolobophora tenuis* Eisen, 1874 [84]: 44.

*Allolobophora subrubicunda* Eisen, 1873 [89]: 51.

*Dendrobaena* (*Dendrodrilus*) *rubida*: Omodeo 1956 [58]: 175.

*Dendrobaena* (*Dendrodrilus*) *rubida* f. *tenuis*: Omodeo 1956 [58]: 175.

*Dendrobaena* (*Dendrodrilus*) *rubida* f. *subrubicunda*: Omodeo 1956 [58]: 175.

*Dendrodrilus rubidus* species complex: Blakemore 2008b [43]: 561 (for complete synonymy).

Diagnosis. Body length 20–90 mm, diameter 2–4 mm. Color red-violet, darker dorsally. Prostomium epilobous, first dorsal pore in 5/6. Setae moderately paired, closer ventrally and wider laterally (Fig 3) Glandular tumescences usually on 9, 16, 22–25 *ab*. Clitellum saddle-shaped, 25, 26–31, 1/n32. Tubercles when present on 29–30 or 28–30. Male pore on 15, equatorial just above setae *b*, on a small porophore confined to its own segment. Female pore on 14 small, dorsad of *b*. Nephropores irregularly alternate between *b* and above *d*. Septa 5/6–10/11 slightly thickened. Calciferous glands in segments 10–12 with large diverticula in segment 10 (Fig 4D). Excretory system holoic. Nephridial bladders U-shaped throughout, with forward-bent ental limbs. Typhlosole well developed lamelliform. The cross-section of longitudinal muscle layer is of pinnate type with slight intermediate feature (Fig 11).

Remarks. *Bimastos rubidus* is a morphologically variable peregrine species with implied parthenogenesis [3]. The *tenuis* morph completely lacks tubercles as well as spermathecae. In the *rubidus* form the spermathecae are sometimes present but usually empty and indistinct tubercles can be seen in 29–30. In the *subrubicundus* form, even filled spermathecae can be seen and the tubercles are easily recognized on 28–30. These forms sometimes are regarded as separate species [18], however, our molecular results did not corroborate this treatment.

#### *Bimastos schwerti* Csuzdi & Chang sp. nov

urn:lsid:zoobank.org:act:B6CE0640-0677-4238-920D-822DAF451732.

**Fig 12:**
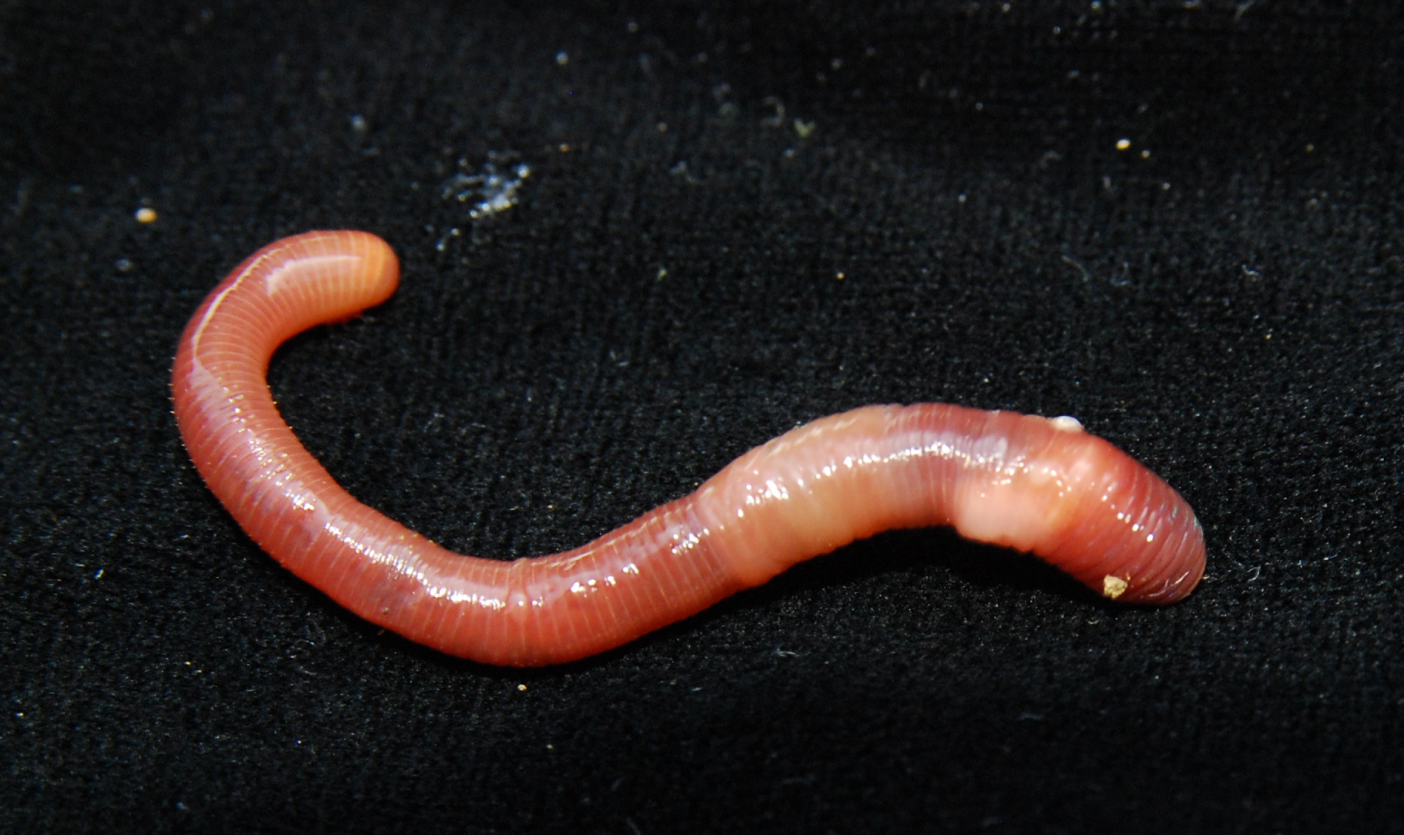
A live specimen of *B*. *schwerti* sp. nov. collected from Siddonsburg, dorsal view.

**Fig 13:**
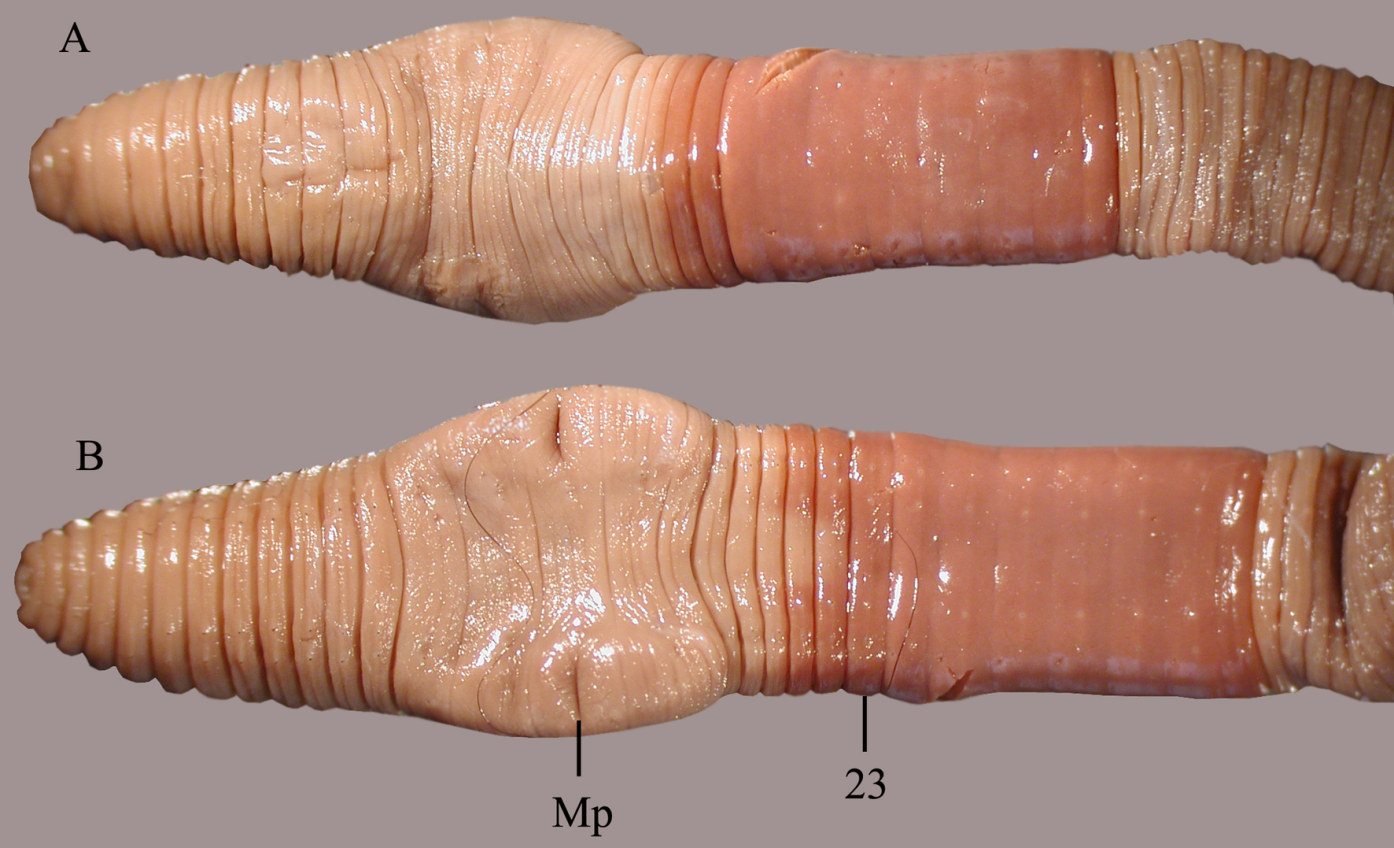
*B*. *schwerti* sp. nov. (Siddonsburg holotype). A = dorsal view, B = ventral view, Mp = male pore.

**Fig 14:**
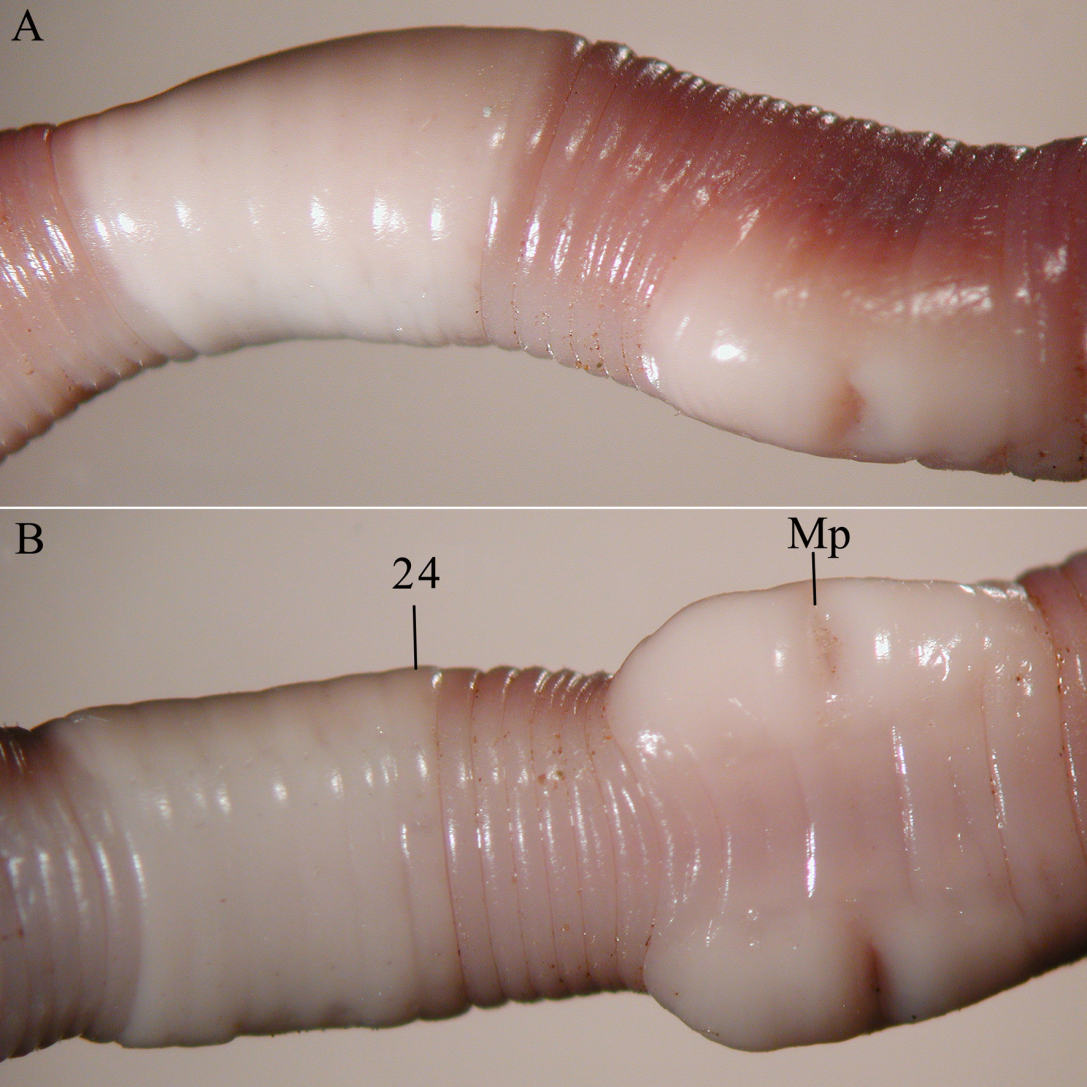
*B*. *schwerti* sp. nov (Jug Bay paratype). A = lateral view of the clitellar region, B = ventral view of the clitellar region, Mp = male pore.

**Fig 15:**
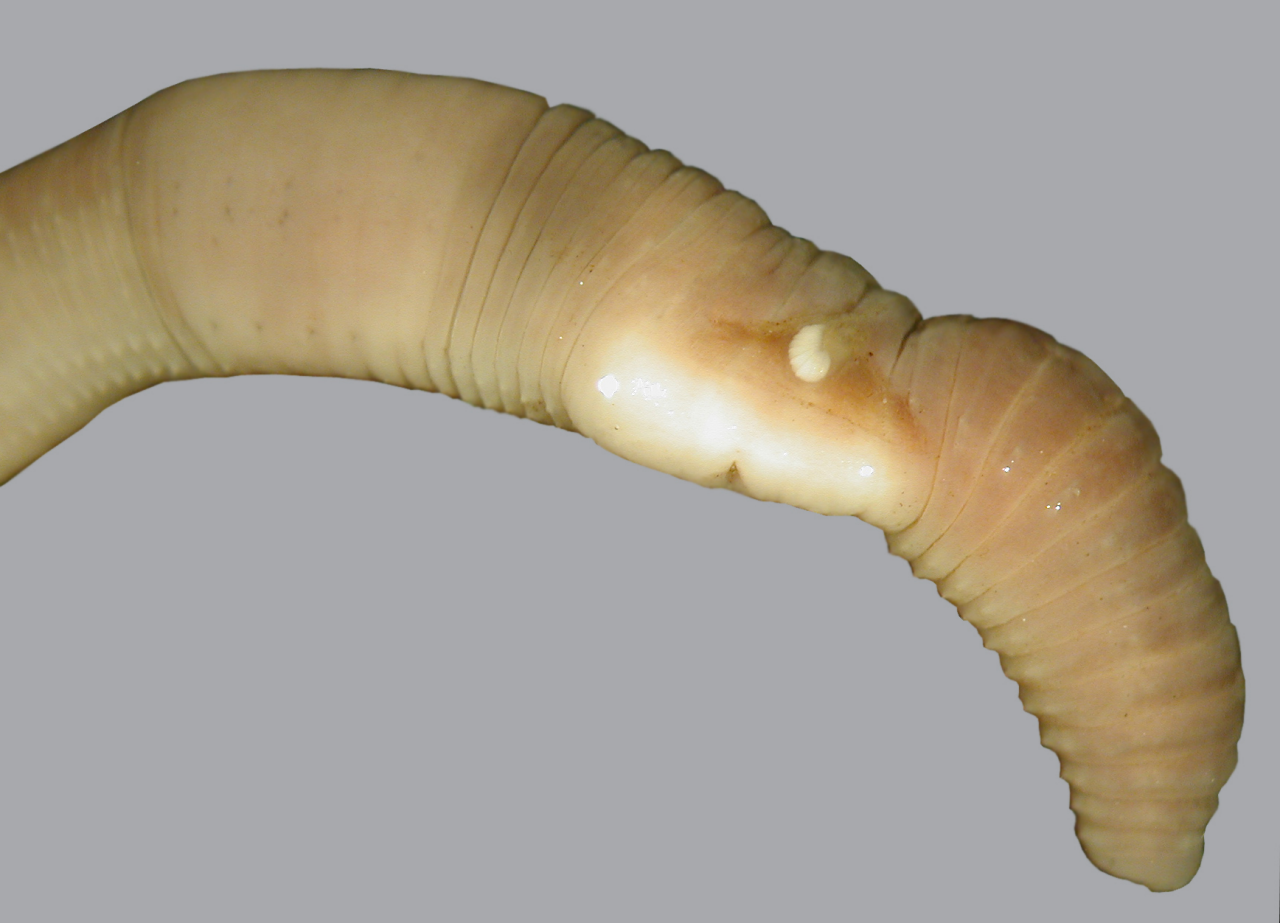
*B*. *schwerti* sp. nov. (Jug Bay paratype). Ventral view of the clitellar region with a flat spermatophore above the male pore.

**Fig 16:**
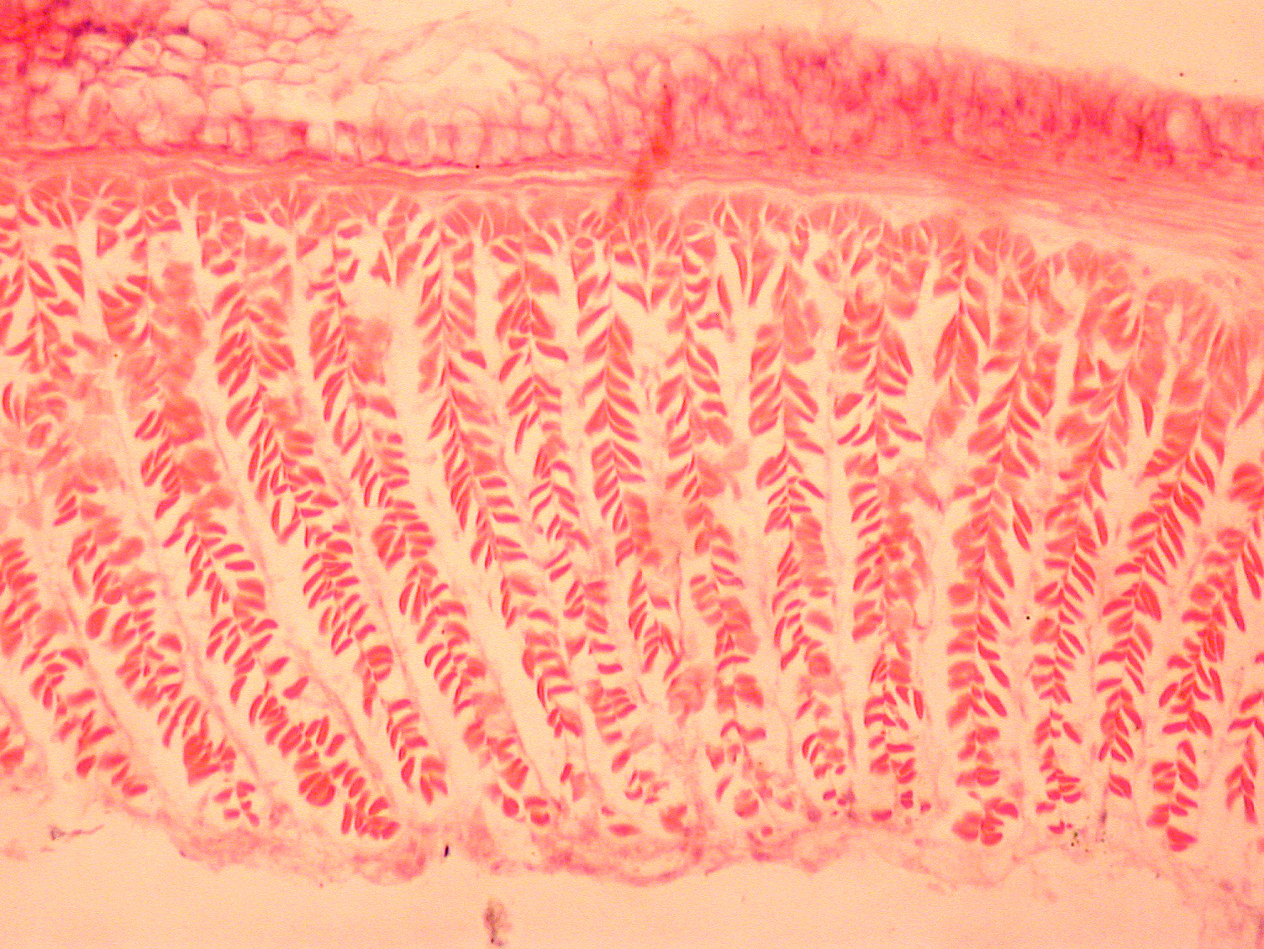
Longitudinal musculature of *B*.*schwerti* (cross section).

Figs 3, 4A and 12–16

Etymology. This species is named in honor of the collector Donald P. Schwert.

Type material. **Holotype,** HNHM/16614, State Game-lands 7 km S of Siddonsburg, York Co., PA, USA (40.10° N; 76.95° W). Leg. D. P. Schwert, 17. 04. 1977. **Paratypes**, HNHM/16615, 10 ex., State Gamelands 7 km. S of Siddonsburg York Co. PA, USA. Leg. D. P. Schwert, 17. 04. 1977. HNHM/14184, 11 ex. Jug Bay Wetlands Sanctuary, Anne Arundel Co. MD, USA. 27. 04. 2001. Leg. Cs. Csuzdi and K. Szlávecz. HNHM/14188, 8 ex., Jug Bay Wetlands Sanctuary, Anne Arundel Co. MD, USA. 27. 04. 2001. Leg. Cs. Csuzdi and K. Szlávecz. HNHM/16500, 2 ex. Jug Bay Wetlands Sanctuary, Anne Arundel Co. MD, USA. 17.05.2012. Leg. Cs. Csuzdi, K. Szlavecz and Ch-H. Chang. HNHM/16510, 3 ex. Jug Bay Wetlands Sanctuary, Anne Arundel Co. MD, USA. 22.06.2007. Leg. K. Szlávecz. HNHM/16566, 3 ex. Jug Bay Wetlands Sanctuary, Anne Arundel Co. MD, USA. 19.04.2003. Leg. K. Szlávecz and S. Pitz. HNHM/16567, 1 ex. Jug Bay Wetlands Sanctuary, Anne Arundel Co. MD, USA. 12.06.2003. Leg. K. Szlávecz. HNHM/17158, 1 ex. Gameland 242, Sidddonsburg, PA, USA. 23.04.2013. Leg. Ch-H- Chang, K. Szlávecz and M. Bernard.

Diagnosis. Body length 25–62 mm, diameter 2–5 mm. Color red-violet (Fig 12). Prostomium epilobous, first dorsal pore in 5/6. Setae moderately paired, glandular tumescences lacking. Clitellum annular, on segments 21, 22, 23, ½23–½30, 30. Male pores on 15, equatorial, just above setae *b*, on extremely large porophores. Female pores on 14 small, slightly dorsad of *b*. Nephropores irregularly alternate between *b* and above *d*. Septa 6/7–8/9 and 12/12–14/15 strengthened. Calciferous glands in segments 10–13 with small diverticula in segment 10 (Fig 4A). Excretory system holoic. Nephridial bladders with forward-bent ental limbs. Typhlosole bifid, the cross-section of longitudinal muscle layer is of pinnate type.

Description. Length of the holotype 62 mm, diameter just after the clitellum 5 mm. Number of segments 117. Paratypes 25–62 mm long and 2–5 mm wide. Number of segments 88–120. Color preserved brown, alive dark red-violet (Fig 12). Prostomium epilobous 1/3 open. First dorsal pore in the intersegmental furrow 5/6. Setae *ab* moderately *cd* more closely paired (Fig 3). Setal formula at segment 35; aa:ab:bc:cd:dd = 5:2:5:1:10. Male pores prominent, facing ventrad on the segment 15 surrounded by huge genital crescents stretching on segments 14–17 (Fig 14). Above the male pore an oval glandular field can be seen similar in structure to the tubercles sometimes with attached flat spermatophore (Fig 15). Nephridiopores irregularly alternating between setal lines *b* and above *d*. Clitellum on 23–30 annular, evenly developed also on the ventral side. In some specimens also 21 and 22 possess tumidity (Fig 13). Tubercula pubertatis lacking. Genital papillae around setae 13–16 *ab* and *cd*. Genital setae S-shaped ca. 1.3 mm long and 0.03 mm wide. Septa 6/7–8/9 and 12/13–14/15 moderately, 9/10–10/11 slightly thickened. Testes and funnels paired in segments 10, 11 surrounded by perioesophageal testis sacs. Two pairs of vesicles prominent in segments 11 and 12. Corresponding to the genital crescents surrounding the male pores large glandular pads appear ventrally on 13–17 and laterally on 13–16. Calciferous glands in 10–13 with small diverticula in 10. Paired hearts appear in segments 7–11 and a pair of small extraoesophageal vessel in 12. Nephridial bladders up to segment 7 hooked proclinate behind clitellum by fusing the two limbs becoming inverted ocarina-shaped or sausage-shaped. Crop in segments 15–16 large saccular, gizzard in segments 17–18 moderately muscular. Typhlosole appears on segment 23 gradually increasing to large, bifid organ filling up 1/4 of the intestine. Longitudinal muscle layer of pinnate type (Fig 16).

Remarks. *Bimastos schwerti* sp. nov. is the second species after *B*. *palustris* with completely developed annular clitellum but differs from it in the position of the clitellum (21,22,23-30 vs. 23–28), the structure of the male pore, and setal arrangement. Between the two populations (Siddonsburg, Pennsylvania and Jug Bay Wetlands Sanctuary, Lothian, Maryland) there are some slight morphological differences. The specimens collected in Maryland are smaller (25–42 mm) and neither of the specimens collected show tumidity in segment 21 or 22. At the Pennsylvania site, *B*. *schwerti* co-occurs with *B*. *longicinctus*, *Aporrectodea caliginosa*, *Ap*. *trapezoides*, *Ap*. *rosea*, *Lumbricus rubellus*, *Octolasion lacteum*, and *Eisenoides carolinensis* (Chang & Szlavecz, personal observation). In the Maryland site it co-occurs with *B*. *heimburgeri*, *B*. *palustris*, *B*. *rubidus*, *Ei*. *lonnbergi*, *L*. *rubellus*, *Eis*. *tetraedra*, *Diplocardia patuxentis*, *Di*. *texensis*.

#### *Bimastos tumidus* (Eisen, 1874)

Fig 3 and 17

**Fig 17:**
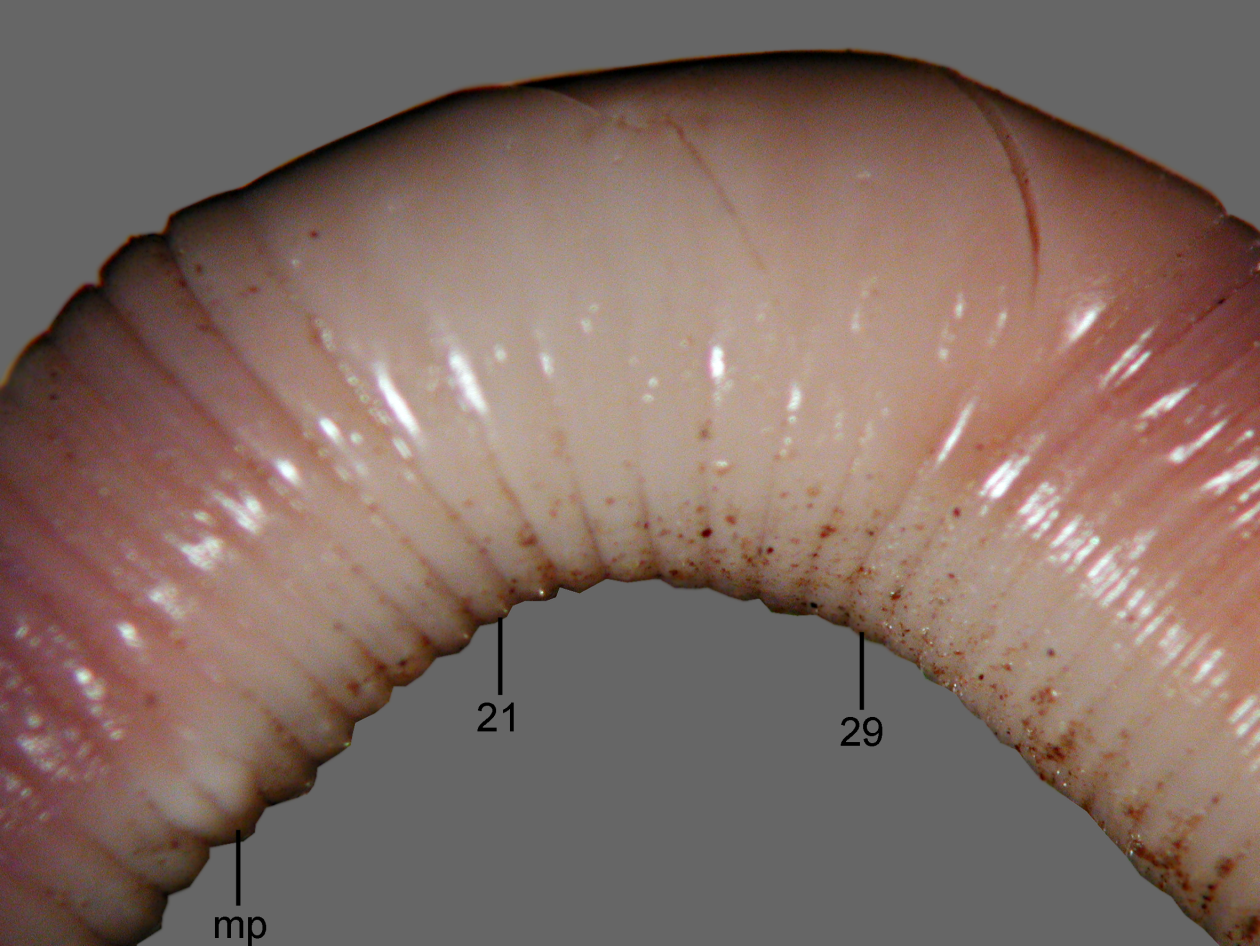
*B*. *tumidus*, lateral view of the clitellar region, mp = male pore, numbers refer to segments.

*Allolobophora tumida* Eisen, 1874 [84]: 45.

*Allolobophora (Bimastus) tumida*: Michaelsen 1899 [59]: 16.

*Allolobophora (Bimastus) gieseleri*: Michaelsen 1899 [59]: 16.

*Helodrilus* (*Bimastus*) *tumidus*: Michaelsen 1900 [10]: 502; Smith 1917 [60]: 170.

*Helodrilus* (*Bimastus*) *gieseleri* var. *hempeli* Smith, 1915 [83]: 551; Smith 1917 [60]: 172.

*Bimastos ducis* Stephenson, 1933 [90]: 939; Gates 1942 [51]: 103.

*Bimastos tumidus*: Gates 1942 [51]: 103; 1956 [65]: 1 (partim); 1969 [5]: 306; Reynolds *et al*. 1974 [81]: 28; Gates 1982 [54]: 27; Zicsi 1981 [13]: 433; Schwert 1990 [79]: 353; Reynolds and Wetzel 2004 [77]: 83; Blakemore 2008a [78]: 6; 2008b [43]: 542.

Material examined. USNM 1164 *Holotype*. Mount Lebanon, California, USA. Leg. G. Eisen (half of sagittally sectioned anterior end with clitellum). USNM 123878, 10 ex. Thomasville, Grady County, Georgia, USA. 04. 02. 1972. Leg. E. Komarek. USNM 123889, 17 ex. Broaddus, San Augustine County, Texas, USA. 08. 08. 1968. Leg. W. Baker, P. Jinright. *Bimastos gieseleri* USNM 25848, 1 ex. Florida, USA. 03. 1896. Leg. A. Hempel. *B*. *gieseleri hempeli* USNM 19683, 20 ex. Guadalupe River, on the bottom, Victoria County, Texas, USA. 25. 04. 1914. Leg. J. D. Mitchell. New records: HNHM/14193, 2 ex. Cross Keys, Baltimore, MD, USA. 24. 04.2001. Leg. K. Szlávecz, Cs. Csuzdi. HNHM/14198, 1 ex. Stony Run stream bank, JHU Homewood Campus, Baltimore, MD, USA. 30. 04.2001. Leg. K. Szlávecz, Cs. Csuzdi. HNHM/16503, 2 ex. Smithsonian Environmental Research Center, Edgewater, MD, USA. 18. 05. 2012. Leg. K. Szlavecz, Cs. Csuzdi. HNHM/16497, 1 ex. Gunpowder Falls State Park, Baltimore Co. MD, USA. 19. 05. 2012. Leg. K. Szlávecz, Cs. Csuzdi.

Diagnosis. Body length 30–40 mm, diameter 2.5–3 mm. Color red-violet dorsally and paler ventrally. Prostomium epilobous, first dorsal pore in 5/6. Setae moderately paired (Fig 3), glandular tumescences lacking. Clitellum saddle-shaped, ½21, 22–29, ½30. Male pore on 15, equatorial just above setae *b*, on a small porophore confined to its own segment (Fig 17). Female pore on 14 small, dorsad of *b*. Nephropores irregularly alternate between *b* and above *d*. No septa notably thickened. Calciferous glands in segments 10–12 with small diverticula in segment 10. Excretory system holoic. Nephridial bladders U-shaped throughout, with forward-bent ental limbs. Typhlosole well developed lamelliform. The cross-section of longitudinal muscle layer is of pinnate type.

Remarks. Gates [65] put *B*. *gieseleri* (Ude, 1985) and *B*. *gieseleri hempeli* (Smith, 1915) in synonymy of *B*. *tumidus*. This action was generally accepted in case of *B*. *gieseleri hempeli* [43, 54, 75, 81]. However most of the authors [54, 75, 78, 79, 81] keep *B*. *gieseleri* separate.

#### *Bimastos welchi* (Smith, 1917)

*Helodrilus welchi* Smith, 1917 [60]: 174.

*Bimastos welchi*: Gates 1942 [51]: 103; 1969 [5]: 306; 1982 [54]: 27; Schwert 1990 [79]: 353; Reynolds and Wetzel 2004 [77]: 83; Blakemore 2008a [78]: 6; 2008b [43]: 537.

Material examined. USNM 16782 *Holotype*. Manhattan, Kansas, USA. 02. 04. 1914. Leg. P.S. Welch.

Diagnosis. Body length 135 mm, diameter 4.5 mm. Color pale throughout. Prostomium epilobous, first dorsal pore in 5/6. Setae closely paired (Fig 3), glandular tumescences lacking. Clitellum almost annular, ventrally less developed on ½25–35. Male pore on 15, equatorial just above setae *b*. Female pore on 14 small, dorsad of *b*. Nephropores irregularly alternate between *b* and above *d*. Septa 6/7–7/8, 12/13 slightly, 8/9–11/12 strongly thickened. Calciferous glands in segments 10–12 with small diverticula in segment 10. Excretory system holoic. Nephridial bladders U-shaped throughout, with forward-bent ental limbs. There is no data on the structure of the longitudinal musculature.

#### *Bimastos zeteki* (Smith and Gittins, 1915)

*Helodrilus (Bimastus) zeteki* Smith and Gittins, 1915 [83]: 545; Smith 1917 [60]: 175.

*Bimastos zeteki*: Gates 1942 [51]: 103; 1969 [5]: 306; Reynolds *et al*. 1974 [81]: 29; Zicsi 1981 [13]: 433; Gates 1982 [54]: 27; Schwert 1990 [79]: 353; Reynolds and Wetzel 2004 [77]: 83; Blakemore 2008a [78]: 6; 2008b [43]: 537.

Material examined. USNM 16782 *Holotype*. Under rotten log, Urbana, Cottonwood Woods, Crossing, Illinois, USA. 25. 03. 1911. Leg. J. Zetek. USNM 26214, 3 ex. Culver, Indiana, USA. 19. 06. 1914. Leg. H.V. Heimburger. USNM 123897, 2 ex. Addison, W of town, Winston Co., Alabama, USA. 17. 04. 1970. Leg. E. Komarek. USNM 123898, 2 ex. Double Springs, Winston Co., Alabama, USA. 18. 04. 1970. Leg. E. Komarek.

Diagnosis. Body length 100–140 mm, diameter 5–6 mm. Color dark red-violet anterio-dorsally and paler ventrally. Prostomium epilobous, first dorsal pore in 5/6. Setae closely paired (Fig 3), glandular tumescences lacking. Clitellum saddle-shaped with slight ventral development on ½27, 27–37. Male pore on 15, equatorial just above setae *b* on a small porophore confined to its own segment. Female pore on 14 small, dorsad of *b*. Nephropores irregularly alternate between *b* and above *d*. Septa 7/8–12/13 slightly, 13/14–14/15 moderately thickened. Calciferous glands in segments 10–12 with small diverticula in segment 10. Excretory system holoic. Nephridial bladders U-shaped throughout, with forward-bent ental limbs. Typhlosole large, lamelliform. There is no data on the structure of the longitudinal musculature.

#### Key to the species of the genus *Bimastos* Moore, 1893

**Table:** 

**1.**	Clitellum annular evenly developed also on the ventral side	**2**
	Clitellum saddle shaped, ventrally always less developed	**3**
**2.**	Male pore postsetal, clitellum on 23-28	***B*. *palustris*** Moore, 1895
	Male pore setal on a huge porophore, clitellum ends on 30	***B*. *schwerti*** sp. nov.
**3.**	Clitellum ends on or after segment 34	**4**
	Clitellum ends on or before segment 33	**6**
**4.**	Clitellum on 24, 25-34, 35	**5**
	Clitellum on 27-37	***B*. *zeteki*** (Smith & Gittins, 1915)
**5.**	Septa 8/9-11/12 thickened, setal ratio *ab*:*cd* = 1.5:1	***B*. *welchi*** (Smith, 1917)
	Septa 12/13-14/15 thickened, setal ratio *ab*:*cd* = 1:1	***B*. *lawrenceae*** Fender, 1994
**6.**	Clitellum ends before segment 31	**7**
	Clitellum ends on or after 31	**9**
**7.**	Septa 7/8-9/10 thickened, typhlosole bifid	***B*. *gieseleri*** (Ude, 1895)
	All septa membranous, Typhlosole lamellar	**8**
**8.**	Clitellum on 24 (25) -30, color dark brown-red	***B*. *parvus*** (Eisen, 1874)
	Clitellum on ½21, 22-29, ½30, color light red-violet	***B*. *tumidus*** (Eisen, 1874)
**9.**	Septa strongly 7/8-11/12 thickened, clitellum on 22, 23-32, 33	***B*. *longicinctus*** (Smith & Gittins, 1915)
	All septa membranous or just slightly thickened	**10**
**10.**	Prostomium tanylobic	***B*. *eiseni*** (Levinsen, 1884)
	Prostomium epilobic	**11**
**11.**	Clitellum on ½24, 25-32, ½33	***B*. *heimburgeri*** (Smith, 1928)
	Clitellum on 26-31, ½23	***B*. *rubidus*** (Savigny, 1826)

## Discussion

Our phylogenetic analyses showed that the genus *Bimastos* as revised in this study is monophyletic and also includes the former *Allolobophoridella eiseni* and *Dendrodrilus rubidus*. Largely in agreement with Gates [5], the revised *Bimastos* is characterized by shared character states that include proclinate U-shaped nephridial bladders, the presence of calciferous diverticula in 10, and having porphyrin-based red pigments. Contrary to Zicsi [13] and Zicsi and Michalis [15], the genera *Spermophorodrilus* and *Healyella* are not related to *Bimastos*. They form a clade nested among several *Dendrobaena* species, and can be distinguished morphologically from *Bimastos* by their sausage-shaped nephridial bladders.

Close relationships among *Bimastos*, *Allolobophoridella eiseni* and *Dendrodrilus rubidus* have been consistently revealed in phylogenetic analyses based on DNA sequences [19–21]. With DNA data comparable in length (5715 bp) to that in Dominguez et al. [21] (5866 bp) while having extensive sampling of *Bimastos* for the first time, our results corroborated previous molecular results and led to our taxonomic treatment that both *Allolobophoridella eiseni* and *Dendrodrilus rubidus* indeed belong to the Nearctic genus *Bimastos*. Although *B*. *eiseni* and *B*. *rubidus* have been exclusively referred to as “European” and considered non-native in North America [9, 55, 91], there has been no genetic evidence suggesting that the two species are truly of European origin. Based on our phylogenetic analyses and the fact that the two species are also reported in North America, of which one is widespread [3, 43, 78, 91], we herein propose that *B*. *eiseni* and *B*. *rubidus*, like *B*. *parvus*, are of North American origin and thus non-native in Europe. The discovery of an earthworm cocoon attributed to *B*. *rubidus* from lake sediment dated over 7,000 years old in Ontario, Canada [92] provides additional proof that the species was present thousands of years before Europeans had reached the continent.

*Bimastos* as herein re-defined is the sister group of *Eisenoides* Gates, 1969, the other North American genus in the family Lumbricidae, confirming the ‘*Bimastos*-*Eisenoides* clade that also contains *Allolobophoridella* and *Dendrodrilus* revealed in Dominguez et al. [21]. The two genera, therefore, form a clade that comprises exclusively North American Lumbricidae, suggesting for the first time that North American lumbricids are indeed monophyletic. This relationship is consistent with that presented in Dominguez et al. [21], and is supported by the proclinate U-shaped nephridial bladders shared by both genera.

*Eisenia* is the sister group of the North American clade both in our extensive dataset and in Dominguez et al. [21]. With the exception of a few Central European species (*E*. *lucens*, *E*. *spelaea and E*. *balatonica*), *Eisenia* is primarily a temperate Asian genus originally from Central and Northeast Asia (the Turanian-Far Eastern earthworm domain [93]), whereas the majority of the endemic North American lumbricids are distributed in eastern US [94]. This geographic distribution, an East Palearctic-East Nearctic disjunction, presents a biogeographic challenge that requires proper explanation.

Molecular clock estimation in Dominguez et al. [21] suggested that the North American *Bimastos*-*Eisenoides* clade diverged from the Eurasian *Eisenia* in the Late Cretaceous, about 72.6 (69.2–76.1) mya, providing some insights into several possible biogeographic scenarios. Three land bridges between Eurasia and North America during the Late Cretaceous (100–66 mya) and Paleocene (66–56 mya) have been proposed to explain distributions of fauna and flora across the two continents: Beringia (connecting Siberia and Alaska), the De Geer route (connecting Northern Europe and Northeastern North America), and the Thulean land bridge (connecting Northwestern Europe and Northeasten North America). When the divergence between closely related taxa is dated back to the Late Cretaceous-Paleocene, the majority of present-day East Palearctic-East Nearctic disjunctions in animals have been attributed to the North Atlantic land bridges (i.e. the De Geer and/or Thulean routes) [95], while those involved Beringia took place more recently (e.g. [96–98]). However, recent evidence has shown that the Thulean land bridge existed only around 57 and 56 mya [99], which is later than the estimated time of cladogenesis between *Eisenia* and the North America clade. Meanwhile, Beringia and the De Geer route existed earlier than the Thulean land bridge (Beringia: 100–75 mya, 65.5 mya, 58 mya; De Geer route: 71–63 mya; [95, 99]), providing possible paths for faunal exchange. A viable hypothesis would thus require ancestral range expansion from Eurasia to North America through either Beringia or the De Geer route followed by extinction in western North America or Europe, respectively. The current native distribution of most *Eisenia* (Central Asia and Northeast Asia,) and the existence of Beringia during Late Cretaceous appear to favor the Beringia hypothesis, but dispersal through the De Geer route is logically equally likely and cannot be ruled out. To test these hypotheses requires more extensive sampling of *Eisenia* covering the native range of the genus and rigorous ancestral range reconstruction using likelihood-based evolution models.

The inferred phylogeny also suggested that *Spermophorodrilus* and *Healyella* form a weakly supported clade. As only three out of the 13 described species from the two genera were sampled, we were unable to conclude whether any of the two is monophyletic. In both of our short and long datasets, the *Spermophorodrilus*-*Healyella* clade, together with *Fitzingeria platyura platyura*, was nested among as many as eight *Dendrobaena* species, including the type species *D*. *octaedra*. Therefore, without synonymizing any of the other three genera, *Dendrobaena* as currently defined is at best paraphyletic. This heterogeneous genus has more than 100 described species [4] and, with some species falling outside of the “*Dendrobaena* clade” as revealed in this study, is in urgent need of revision. Our phylogenetic analyses encompassed the type species of all four genera, and from the nomenclature point of view, it is justifiable to synonymize the four names. However, we strongly oppose to do this, because (1) it would lead to losing evolutionarily meaningful morphological information that is currently included in the diagnosis of the respective genera, and (2) it would impede further research towards a comprehensive revision of the genus *Dendrobaena*. We also believe that any nomenclature act regarding the involved genera should only be done along with a full revision of the genus *Dendrobaena*.

The chaos in the systematics of *Bimastos*, *Allolobophoridella*, *Dendrodrilus*, *Spermophorodrilus*, and *Healyella* highlighted the difficulty of understanding character evolution and the respective synapomorphy and autapomorphy in earthworm systematics. The striking morphological similarity among *Bimastos*, *Spermophorodrilus*, and *Healyella* is an example of homoplasy. The annular clitellum was believed to be the defining synapomorphy uniting *Bimastos* and *Healyella*, [13, 15], but it turned out to be acquired independently in the *Bimastos* clade and in the *Spermophorodrilus*-*Healyella* clade. All three genera shifted to phoral dissemination which resulted in disappearance of the spermathecae and tubercles. Disappearance of tubercles made it possible to develop an annular clitellum, which is more appropriate for cocoon formation. However, to facilitate the successful copulation, glandular genital ridges (pseudotubercles) developed in the male pore region [14, 100].

The revised *Bimastos* genus shares proclinate U-shaped nephridial bladders, the presence of calciferous diverticula in 10, and porphyrin based red pigments among its species, while at the same time allows both tanylobous and epilobous prostomium and both pinnate and fasciculated longitudinal musculature in the genus. The presence of tanylobous prostomium, an autapomorphy in the case of *Bimastos*, was part of the reason why *B*. *eiseni* was originally classified as a *Lumbricus* [67], a genus whose species shares tanylobous prostomium as synapomorphy. Similarly, Pop [11] believed that inclusion of more than one type of musculature in a genus renders it polyphyletic. However, two types of musculature can be seen in *Bimastos*, as is also the case in *Eisenia*, in which the musculature of *Eisenia lucens* (Waga, 1857) is fasciculated while the other three species in Dominguez et al.’s [21] monophyletic *Eisenia* genus possess pinnate musculature [3].

The native range of most *Bimastos* species is currently restricted to Eastern North America, with the distribution of some species, such as *Bimastos welchi*, well extending into the Great Plains, states of Kansas and Colorado in the USA [5, 91, 101]. Gates [9] believed that the genus once had a much wider range that had significantly reduced due to glaciations. This hypothesis is supported by the discovery of *Bimastos lawrenceae* Fender, 1994 from Vancouver Island in Northwestern North America, a location far north of the last glacial boundary [56]. The fossil cocoon attributed to *B*. *rubidus* from lake sediment in Ontario, Canada [92] lends further support that *Bimastos* might have covered also the terrain that is now Canada, and its representatives were able to survive, at least in a few favorable refuges, during the Ice Age.

Our understanding of the peregrine *Bimastos eiseni* and *B*. *rubidus*, has been misled by the poor knowledge of the systematics of this genus, especially in Europe, where they have been mistakenly categorized as indigenous for more than a century [3, 9]. The interception of the two species in imported soil at US ports was further viewed, again mistakenly, as direct evidence for introduction to North America [102]. It is now clear that like *B*. *parvus*, both *B*. *eiseni* and *B*. *rubidus* are native to North America, and, in contrast to the commonly held belief, should be categorized as introduced in Europe.

*Bimastos parvus* is considered a peregrine species that also has been intercepted in shipments at North American ports [9]. Similar to other peregrine earthworms, the three *Bimastos* species were likely to be transported back and forth between continents and even today can easily enter into countries without strict regulations on soil, plants and timber import. Both *B*. *eiseni* and *B*. *rubidus* have been found in different types of wetlands, along streams, and under bark of trees, rotting logs, pile of leaf litter, compost, moss, and dung [55, 103]. It appears that cool, moist, sometimes acidic habitats with high organic matter content are optimal for these species [55]. This might explain the occurrence of *B*. *rubidus* at high elevations in the tropics [9, 43] and at high latitudes, although temperature may still limit their distribution if it does not reach the degree days necessary for individual development [104]. It also highlights why they might travel well, as they were often intercepted in potted plants covered with *Sphagnum* moss.

We conclude that the combined morphological, molecular and biogeographical anaysis was necessary to reveal interesting, novel information about the phylogeny and distribution of the *Bimastos* genus. On the one hand, the classical, purely morphology-based taxonomy proved to be wrong in the past; on the other hand, relying on DNA analysis alone is insufficient to define genera. It is our hope that such integrated approach will lead to a more consistent system of Lumbricidae.

The study also highlights that not all peregrine lumbricid species in North America came from Europe; reverse introduction also occurred. Additionally, our results generated exciting biogeographical hypotheses to be tested in the future.

## Supporting information

S1 Table Species from Domínguez et al. (2015) and Pérez-Losada et al. (2015) used in this study and their GenBank accession numbers or voucher numbers.
(DOCX)

S2 Table Primers used in polymerase chain reactions (PCR). (DOCX)

## References

[1] Lee KE. Earthworms: their ecology and relationships with soils and land use Sydney: Academic Press; 1985. 411 p.

[2] Zicsi A, Csuzdi C. Weitere Angaben zur Regenwurmfauna Frankreichs mit Beschreibung fünf neuer Arten (Oligochaeta: Lumbricidae). Revue suisse de Zoologie. 1999;106:983−1003.

[3] Csuzdi C, Zicsi A. Earthworm of Hungary. Budepast: Hungarian Natural History Museum; 2003. 271 p.

[4] Csuzdi C. Earthworm species, a searchable database. Opuscula Zoologica Budapest. 2012;43(1):97−9.

[5] Gates GE. On two American genera of the earthworm family Lumbricidae. Journal of Natural History. 1969;9:305−7.

[6] Reynolds JW. The distribution of the earthworms (Oligochaeta) of Indiana: A case for the post quaternary introduction theory for megadrile migration in North America. Megadrilogica. 1994;5(3):13−32.

[7] Szlavecz K, Csuzdi C. Land use change affects earthworm communities in Eastern Maryland, USA. European Journal of Soil Biology. 2007;43:S79−S85. doi: 10.1016/j.ejsobi.2007.08.008

[8] Chang CH, Szlavecz K, Filley T, Buyer JS, Bernard MJ, Pitz SL. Belowground competition among invading detritivores. Ecology. 2016;97(1):160−70. doi: 10.1890/15-0551.1 27008785 10.1890/15-0551.1

[9] Gates GE. Burmese earthworms—Introduction to systematics and biology of megadrile oligochaetes with special reference to Southeast Asia. Transactions of the American Philosophical Society. 1972;62:5–324.

[10] Michaelsen W. Das Tierreich 10, Oligochaeta. Berlin: R. Friedländer und Sohn; 1900. 575 p.

[11] Pop V. Zur phylogenie und systematik der lumbriciden. Zoologische Jahrbücher Abteilung für Systematik Ökologie und Geographie der Tiere. 1941;74:487−522.

[12] Perel TS. A critical analysis of the Lumbricidae genera system (with key to the USSR fauna genera). Revue D'écologie et de Biologie du Sol. 1976;13:635−43.

[13] Zicsi A. Probleme der Lumbriciden-Systematik sowie die Revision zweier Gattungen (Oligochaeta). Acta Zoologica Hungarica. 1981;27:431−42.

[14] Omodeo P, Rota E. Earthworms of Turkey. Italian Journal of Zoology. 1989;56:167−99.

[15] Zicsi A, Michalis K. Zwei neue Dendrobaena-Arten aus Grichenland (Oligochaeta: Lumbricidae). Acta Zoologica Hungarica. 1993;39:301−10.

[16] Muldal S. A new species of earthworm of the genus *Allolobophora*. Proceedings of the Zoological Society of London. 1952;122:463−5.

[17] Mršić N. Monographs of Earthworms on the Balkans I-II. Ljubljana: SAZU; 1991. 757 p.

[18] Qiu JP, Bouché M. Classified list of earthworm (Oligochaeta: Lumbricoidea) after the study of the three fifth of them. Documents Pedozoologiques & Integrologiques. 1998;4:181−200.

[19] Cech G, Csuzdi C, Marialigeti K. Remarks on the molecular phylogeny of the genus *Dendrobaena* (sensu Pop 1941) based on the investigation of 18S rDNA sequences. Advances in earthworm taxonomy II (Annelida: Oligochaeta). 2005:85−98.

[20] Pop AA, Cech G, Wink M, Csuzdi C, Pop VV. Application of 16S, 18S rDNA and COI sequences in the molecular systematics of the earthworm family Lumbricidae (Annelida, Oligochaeta). European Journal of Soil Biology. 2007;43:S43−S52. doi: 10.1016/j.ejsobi.2007.08.007

[21] Dominguez J, Aira M, Breinholt JW, Stojanovic M, James SW, Perez-Losada M. Underground evolution: New roots for the old tree of lumbricid earthworms. Molecular Phylogenetics and Evolution. 2015;83:7−19. doi: 10.1016/j.ympev.2014.10.024 25463017 10.1016/j.ympev.2014.10.024 PMC4766815

[22] Zicsi A. Über die Regenwürmer Ungarns (Oligochaeta Lumbricidae) mit Bestimmungstabellen der Arten. Opuscula Zoologica Budapest. 1991;24:167–91.

[23] Csuzdi C. Towards a phylogenetic concept of Lumbricid systematics In: Moreno AG, Borges S, editors. Advances in earthworm taxonomy (Annelida: Oligochaeta). Madrid: Editorial Complutense; 2004 p. 333−46.

[24] Chang C-H, Lin S-M, Chen J-H. Molecular systematics and phylogeography of the gigantic earthworms of the *Metaphire formosae* species group (Clitellata, Megascolecidae). Molecular Phylogenetics and Evolution. 2008;49(3):958−68. doi: 10.1016/j.ympev.2008.08.025 18809504 10.1016/j.ympev.2008.08.025

[25] Chang C-H, James S. A critique of earthworm molecular phylogenetics. Pedobiologia. 2011;54:S3−S9. doi: 10.1016/j.pedobi.2011.07.015

[26] Perez-Losada M, Breinholt JW, Porto PG, Aira M, Dominguez J. An earthworm riddle: Systematics and phylogeography of the Spanish lumbricid *Postandrilus*. Plos One. 2011;6(11). doi: 10.1371/journal.pone.0028153 22140529 10.1371/journal.pone.0028153 PMC3226679

[27] Fernandez R, Almodovar A, Novo M, Simancas B, Diaz Cosin DJ. Adding complexity to the complex: New insights into the phylogeny, diversification and origin of parthenogenesis in the *Aporrectodea caliginosa* species complex (Oligochaeta, Lumbricidae). Molecular Phylogenetics and Evolution. 2012;64(2):368−79. doi: 10.1016/j.ympev.2012.04.011 22542691 10.1016/j.ympev.2012.04.011

[28] Novo M, Fernandez R, Fernandez Marchan D, Trigo D, Diaz Cosin DJ, Giribet G. Unearthing the historical biogeography of Mediterranean earthworms (Annelida: Hormogastridae). Journal of Biogeography. 2015;42(4):751−62. doi: 10.1111/jbi.12447

[29] Raw F. Estimating earthworm populations by using formalin. Nature. 1959;184:1661–2.

[30] Krutsay M. Histological technics Budapest: Medicina Press; 1980. 202 p.

[31] Perez-Losada M, Breinholt JW, Aira M, Dominguez J. An updated multilocus phylogeny of the Lumbricidae (Annelida: Clitellata: Oligochaeta) earthworms. Journal of Phylogenetics and Evolutionary Biology. 2015;3(1):140.

[32] Katoh K, Standley DM. MAFFT Multiple Sequence Alignment Software Version 7: Improvements in Performance and Usability. Molecular Biology and Evolution. 2013;30(4):772−80. doi: 10.1093/molbev/mst010 23329690 10.1093/molbev/mst010 PMC3603318

[33] Xia X. DAMBE5: A comprehensive software package for data analysis in molecular biology and evolution. Molecular Biology and Evolution. 2013;30(7):1720−8. doi: 10.1093/molbev/mst064 23564938 10.1093/molbev/mst064 PMC3684854

[34] Darriba D, Taboada GL, Doallo R, Posada D. jModelTest 2: more models, new heuristics and parallel computing. Nature Methods. 2012;9(8):772. 10.1038/nmeth.2109 PMC4594756 22847109

[35] Stamatakis A. RAxML version 8: a tool for phylogenetic analysis and post-analysis of large phylogenies. Bioinformatics. 2014;30(9):1312−3. doi: 10.1093/bioinformatics/btu033 24451623 10.1093/bioinformatics/btu033 PMC3998144

[36] Miller MA, Pfeiffer W, Schwartz T, editors. The CIPRES science gateway: a community resource for phylogenetic analyses. Proceedings of the 2011 TeraGrid Conference: extreme digital discovery; 2011: ACM.

[37] Ronquist F, Teslenko M, van der Mark P, Ayres DL, Darling A, Hohna S, et al MrBayes 3.2: efficient Bayesian phylogenetic inference and model choice across a large model space. Systematic Biology. 2012;61(3):539−42. doi: 10.1093/sysbio/sys029 22357727 10.1093/sysbio/sys029 PMC3329765

[38] Mršić N. Description of a new subgenus, three new species and taxonomic problems of the genus Allolobophora sensu Mršić & Šapkarev 1988 (Lumbricidae, Oligochaeta). Bioloski vestnik Lubljana. 1990;38:49−68.

[39] Rosa D. Revisione dei lumbricidi. Memoire della Reale Academia delle Scienze di Torino (Serie 2). 1893;43:399–477.

[40] Černosvitov L. Zur Kenntnis der Oligochätenfauna des Balkans. Zoologischer Anzeiger. 1938;123:192−200.

[41] Moore HF. Preliminary account of a new genus of Oligochaeta. Zoologischer Anzeiger. 1893;16:333−4.

[42] Zicsi A, Michalis K. Übersicht der Regenwurm-fauna Griechenlands (Oligochaeta: Lumbricidae). Acta Zoologica Hungarica. 1981;27:239−64.

[43] Blakemore RJ. Cosmopolitan earthworms 3rd ed. Yokohama: VermEcology; 2008. 757 p.

[44] Bouché MB. La reproduction de Spermophorodrilus albanianus nov. gen. nov. sp. (Lumbricidae) explique-t-elle la fonction des spermatophores? Zoologische Jahrbücher Abteilung für Systematik, Geographie und Biologie der Tiere. 1975;102:1−11.

[45] Omodeo P, Rota E. Earthworms of Turkey II. Italian Journal of Zoology. 1991;58:171−81.

[46] Csuzdi C, Zicsi A, Misirlioglu M. An annotated checklist of the earthworm fauna of Turkey (Oligochaeta: Lumbricidae). Zootaxa. 2006;1175:1−29.

[47] Pavlíček T, Csuzdi C, Misirlioglu M, Vilenkin B. Faunistic similarity and endemism of earthworms in east mediterranean region. Biodiversity and Conservation. 2010;19:1989−2001.

[48] Csuzdi C, Pavlícek T. Earthworms from Israel and the neighbouring countries. I. Genera *Dendrobaena* Eisen, 1874 and Bimastos Moore, 1891 (Oligochaeta: Lumbricidae). Israel Journal of Zoology. 1999;45:467−86.

[49] Csuzdi C, Pavlíček T. Murchieona minuscula (Rosa, 1906) a newly recorded earthworm from Israel and disgtribution of the genera *Dendrobaena* and *Bimastos* in Israel (Ologochaeta, Lumbricidae). Zoology in the Middle East. 2002;25:105−14.

[50] Moore HF. On the structure of *Bimastos palustris*, a new Oligochaete. Journal of Morphology. 1895;10:473−96.

[51] Gates GE. Check list and bibliography of North American earthworms. The American Midland Naturalist. 1942;27:86−108.

[52] Gates GE. Contributions to a revision of the earthworm family Lumbricidae XII. *Enterion mammale* Savigny, 1826 and its position in the family. Megadrilogica. 1975;2(1):1−5.

[53] Reynolds JW. The earthworms (Lumbricidae and Sparganophilidae) of Ontario. Toronto: Royal Ontario Museum; 1977. 141 p.

[54] Gates GE. Farewell to North American megadriles. Megadrilogica. 1982;4(1–2):12–77.

[55] Fender WM. Earthworms of the western United States. I. Lumbricidae. Megadrilogica. 1985;4:93−129.

[56] McKey-Fender D, Fender WM, Marshall VG. North American earthworms native to Vancouver Island and the Olympic Peninsula. Canadian Journal of Zoology. 1994;72:1325−39.

[57] Stephenson J. The Oligochaeta. Oxford: Clarendon Press; 1930. 978 p.

[58] Omodeo P. Contributo alla revisione dei Lumbricidae. Archivio Zoologico Italiano. 1956;41:129−212.

[59] Michaelsen W. Die Lumbriciden-fauna Nordamerikas. Abhandlungen und Verhandlungen des Naturwissenschaftlichen Vereins in Hamburg. 1899;16:1−22.

[60] Smith F. North American earthworms of the family Lumbricidae in the collections of the United States National Museum. Proceedings of the United States National Museum. 1917;52(2174):157−82.

[61] Zicsi A. Verzeichnis der bis 1971 beschriebenen und revidierten Taxa der Familie Lumbricidae (Oligochaeta). Acta Zoologica Hungarica. 1982;28:421−54.

[62] Perel TS. Range and regularities in the distribution of earthworms of the USSR fauna Moscow: Nauka; 1979. 272 p.

[63] Perel TS. Structural peculiarities of genital system of the Lumbricidae related to differences in the way of insemination. Zoologicheski Journal. 1980;59(4):507–17.

[64] Qiu JP, Bouché M. The interpretation of earthworm characteristics. Documents Pedozoologiques & Integrologiques. 1998;3:119−78.

[65] Gates GE. Notes on American earthworms of the family Lumbricidae. III-VII. Bulletin of the Museum of Comparative Zoology at Harvard College. 1956;115:1−46.

[66] Reynolds JW. Are oligochaetes really hermaphroditic amphimictic organisms? The Biologist. 1974;56(2):90–9.

[67] Levinsen GMR. Systematisk-geografisk oversigt over de nordiske Annulata, Gephyrea, Chaetognathi og Balanoglossi. Videnskabelige Meddelelser fra den naturhistoriske Forening i Kjöbenhavn. 1884;45:92−384.

[68] Easton EG. A guide to the valid names of earthworms (Oligochaeta) In: Satchell JE, editor. Earthworm ecology from Darwin to vermiculture. London: Chapman and Hall; 1983 p. 475–85.

[69] Bretscher K. Südschweizerische Oligochaeten. Revue suisse de Zoologie. 1900;8:435−59.

[70] Friend H. The distribution of British annelids. Zoologist. 1911;4(15):143–6, 84–91, 367−74.

[71] Pop V. Neue lumbriciden aus Rumänien. Buletinul Societatii de Stiinte din Cluj. 1938;9:134−52.

[72] Pop V. Lumbricidele din România. Analele Academiei Republicii Populare Române. 1949;1(9):383−505.

[73] Zicsi A. Faunistisch-systematische und ökologische Studien über die Regenwürmer Ungarns. I. Acta Zoologica Hungarica. 1959;5:165−89.

[74] Zicsi A. Ein zusammenfassendes Verbreitungsbild der Regenwürmer auf Grund der Boden- und Vegetations verhältnisse Ungarns. Opuscula Zoologica Budapest. 1968;8:99−164.

[75] Reynolds JW. Status of exotic earthworm systematic and biogeography in North America In: Hendrix PF, editor. Earthworm Ecology and Biogeography in North America. Boca Raton: Lewis Publishers; 1995 p. 1−27.

[76] Ude H. Über zwei neue Lumbriciden-Arten aus Nordamerika. Zoologischer Anzeiger. 1895;18:339.

[77] Reynolds JW, Wetzel MJ. Terrestrial oligochaeta (Annelida: Clitellata) in North America north of Mexico. Megadrilogica. 2004;9(11):71–98.

[78] Blakemore RJ. American earthworms from north of the Rio Grande- a species checklist. 3rd ed2008.

[79] Schwert DP. Oligochaeta: Lumbricidae In: Dindal DL, editor. Soil Biology Guide. New York: Wiley and Sons; 1990 p. 341−56.

[80] Smith F. An account of changes int he earthworm fauna of Illinois and a description of one new species. Bulletin of the Illinois State Natural History Survey. 1928;17:347−62.

[81] Reynolds JW, Clebsch EC, Reynolds WM. Contribution to North American earthworms (Oligochaeta) no. 13. The earthworms of Tenessee (Oligochaeta) I. Lumbricidae. Bulletin of Tall Timbers Research Station. 1974;17:1−133.

[82] Marshall VG, Fender WM. Native and introduced earthworms (oligochaeta) of British Columbia, Canada. Megadrilogica. 2007;11(4):29−52.

[83] Smith F, Gittins EM. Two new species of Lumbricidae from Illinois. Bulletin of the Illinois State Laboratory of Natural History. 1915;10(7):545−50.

[84] Eisen G. New Englands och Canadas Lumbricider. Öfversigt af Kongliga Vetenskaps-Akademiens Förhandligar. 1874;31(2):41−9.

[85] Michaelsen W. Die regenwurm-fauna von Florida und Georgia. Zoologische Jahrbücher Abteilung für Systematik Geographie und Biologie der Tiere. 1894;8:177−94.

[86] Bouché MB. Lombriciens de France. Écologie et Systématique. Dijon, France: Institut National de la Recherche Agronomique; 1972. 671 p.

[87] Mercadal de Barrio I, Barrio A. Nuevos hallazgos y otras citas para la oligoquetofauna de Argentina. Physis (C). 1988;46(110):1−4.

[88] Savigny JC. In: Cuvier G, editor. Analyse des Travaux de l’Academie royale des Sciences, pendant l’année 1821, partie physique Mémoires de l’Académie des Sciences de l’Institut de France Paris. 5. Paris: Académie des Sciences de l’Institut de France; 1826 p. 176−84.

[89] Eisen G. Om Skandinaviens Lumbricider. Öfversigt af Kongliga Vetenskaps-Akademiens Förhandligar. 1873;30(8):43−56.

[90] Stephenson J. Oligochaeta from Australia, North Carolina, and other parts of the world. Proceedings of the Zoological Society, London. 1933;1932:899−941.

[91] Reynolds JW, Wetzel MJ. Terrestrial Oligochaeta (Annelida: Clitellata) in North America, including Mexico, Puerto Rico, Hawaii, and Bermuda. Megadrilogica. 2008;12(12):157−208.

[92] Schwert DP. Description and significance of a fossil earthworm (Oligochaeta: Lumbricidae) cocoon from postglacial sediments in southern Ontario. Canadian Journal of Zoology. 1979;57(7):1402−5.

[93] Csuzdi C, Pop VV, Pop AA. The earthworm fauna of the Carpathian Basin with new records and description of three new species (Oligochaeta: Lumbricidae). Zoologischer Anzeiger. 2011;250:2−18.

[94] James SW. Systematics, biogeography, and ecology of Nearctic earthworms from Eastern, Central, Southern, and Southwestern United States In: Hendrix PF, editor. Earthworm ecology and biogeography in North America. Boca Raton: Lewis Publishers; 1995 p. 29−51.

[95] Sanmartin I, Enghoff H, Ronquist F. Patterns of animal dispersal, vicariance and diversification in the Holarctic. Biological Journal of the Linnean Society. 2001;73(4):345−90. doi: 10.1006/bijl.2001.0542

[96] Burbrink FT, Lawson R. How and when did Old World ratsnakes disperse into the New World? Molecular Phylogenetics and Evolution. 2007;43(1):173−89. doi: 10.1016/j.ympev.2006.09.009 17113316 10.1016/j.ympev.2006.09.009

[97] Vila R, Bell CD, Macniven R, Goldman-Huertas B, Ree RH, Marshall CR, et al Phylogeny and palaeoecology of *Polyommatus* blue butterflies show Beringia was a climate-regulated gateway to the New World. Proceedings of the Royal Society B-Biological Sciences. 2011;278(1719):2737−44. doi: 10.1098/rspb.2010.2213 21270033 10.1098/rspb.2010.2213 PMC3145179

[98] Wu L-W, Yen S-H, Lees DC, Lu C-C, Yang P-S, Hsu Y-F. Phylogeny and Historical Biogeography of Asian *Pterourus* butterflies (Lepidoptera: Papilionidae): a case of intercontinental dispersal from North America to East Asia. Plos One. 2015;10(10). doi: 10.1371/journal.pone.0140933 26484776 10.1371/journal.pone.0140933 PMC4617649

[99] Brikiatis L. The De Geer, Thulean and Beringia routes: key concepts for understanding early Cenozoic biogeography. Journal of Biogeography. 2014;41(6):1036−54. doi: 10.1111/jbi.12310

[100] Omodeo P. Evolution and biogeography of megadriles (Annelida, Clitellata). Italian Journal of Zoology. 2000;67:179–201.

[101] Reynolds JW, Wetzel MJ. Terrestrial Oligochaeta (Annelida: Clitellata) in North America, including Mexico, Puerto Rico, Hawaii, and Bermuda. III. Megadrilogica. 2012;15(8):191–211.

[102] Gates GE. More on oligochaete distribution in North America. Megadrilogica. 1976;2(11):1−8.

[103] Gerard BM. British Lumbricidae. Synopses of the British Fauna No. 6. London: The Linnean Society; 1964. 58 p.

[104] Berman DI, Marusik YM. On Bimastos parvus (Oligochaeta: Lumbricidae) from Yukon Territory (Canada), with discussion of distribution of the earthworms in northwestern North America and northeastern Siberia. Megadrilogica. 1994;5:113−6.

