# Supplementary material for: Molecular phylogeny and systematics of native North American lumbricid earthworms (Clitellata: Megadrili)
Source: PLoS One. 2017 Aug 9;12(8):e0181504. doi: 10.1371/journal.pone.0181504 (PMC5549934; doi:10.1371/journal.pone.0181504)
Supplement: S1 Table — (2015) and Pérez-Losada et al. (2015) used in this study and their GenBank accession numbers or voucher numbers. (DOCX) [file pone.0181504.s001.docx]

S1 Table. Species from Domínguez et al. (2015) and Pérez-Losada et al. (2015) used in this study and their GenBank accession numbers or voucher numbers.

| **Species** | **12S** | **16S** | **18S** | **COI** | **COII** | **ND1** |
| --- | --- | --- | --- | --- | --- | --- |
| **Family Hormogastridae** |  |  |  |  |  |  |
| *Hormogaster castillana* | KJ912489 | KJ912603 | KJ912026 | MPL480 | KJ912142 | KJ912367 |
| *Hormogaster elisae* | KJ912488 | KJ912602 | KJ912025 | na | KJ912141 | KJ912366 |
|  |  |  |  |  |  |  |
| **Family Lumbricidae** |  |  |  |  |  |  |
| *Allolobophora chlorotica* | KJ912384 | KJ912505 | KJ911922 | MPL418 | KJ912048 | KJ912274 |
| *Allolobophora dacica* | KJ912383 | KJ912504 | KJ911921 | MPL336 | KJ912047 | KJ912273 |
| *Alpodinardella gestroi* | KJ912439 | na | KJ911985 | MPL427 | KJ912096 | KJ912277 |
| *Aporrectodea jassyensis* | na | KJ912510 | KJ911927 | MPL404 | na | KJ912279 |
| *Aporrectodea limicola* | FJ967195 | FJ967636 | KJ911930 | JN850541 | na | FJ967318 |
| *Aporrectodea morenoe* | na | JN872090 | KJ911934 | MPL348 | JN871996 | JN872136 |
| *Aporrectodea trapezoides* | FJ967187 | FJ967627 | KJ911940 | MPL156 | na | FJ967310 |
| *Bimastos welchi* | KJ912396 | KJ912520 | KJ911943 | na | KJ912057 | KJ912286 |
| *Bimastos zeteki* | na | KJ912521 | KJ911944 | na | KJ912058 | na |
| *Cataladrilus monticola* | JN872050 | JN872095 | KJ911946 | MPL315 | JN872001 | JN872137 |
| *Cernosvitovia dudichi* | KJ912397 | na | KJ911948 | MPL417 | KJ912059 | KJ912287 |
| *Dendrobaena illyrica* | KJ912402 | KJ912524 | KJ911954 | MPL425 | na | KJ912291 |
| *Dendrobaena pentheri* | KJ912407 | na | KJ911959 | MPL406 | na | KJ912295 |
| *Dendrobaena veneta* | KJ912413 | na | KJ911963 | MPL433 | KJ912070 | KJ912298 |
| *Diporodrilus pilosus* | KJ912423 | KJ912542 | KJ911971 | MPL357 | KJ912078 | KJ912307 |
| *Eisenia balatonica* | KJ912425 | KJ912544 | KJ911973 | MPL324 | KJ912080 | KJ912309 |
| *Eisenia lucens* | KJ912430 | KJ912549 | KJ911978 | MPL421 | KJ912085 | KJ912314 |
| *Eiseniella tetraedra* | KJ912433 | KJ912553 | KJ911979 | MPL347 | KJ912091 | KJ912317 |
| *Eiseniona albolineata* | KJ912434 | KJ912554 | KJ911980 | MPL250 | KJ912092 | KJ912318 |
| *Eiseniona oliveirae* | KJ912435 | KJ912555 | KJ911981 | MPL251 | KJ912093 | KJ912319 |
| *Helodrilus cernosvitovianus* | KJ912440 | KJ912562 | KJ911988 | MPL332 | KJ912098 | KJ912323 |
| *Lumbricus castaneus* | KJ912445 | KJ912566 | KJ911993 | MPL372 | KJ912099 | KJ912325 |
| *Octodrilus transpadanus* | KJ912457 | KJ912576 | KJ912003 | MPL420 | KJ912111 | KJ912337 |
| *Panoniona leoni* | KJ912466 | KJ912584 | KJ912011 | MPL410 | KJ912119 | KJ912346 |
| *Postandrilus majorcanus* | JN872014 | JN872063 | KJ912042 | MPL378 | JN871962 | JN872108 |
| *Proctodrilus antipai* | na | KJ912585 | KJ912012 | MPL333 | KJ912120 | KJ912347 |
| *Prosellodrilus pyrenaicus* | JN872053 | JN872097 | KJ912015 | MPL320 | JN872004 | JN872139 |
| *Scherotheca corsicana* | KJ912470 | KJ912587 | KJ912017 | MPL358 | KJ912123 | KJ912349 |
| *Scherotheca gigas* | KJ912471 | KJ912588 | KJ912018 | MPL212 | KJ912124 | KJ912350 |
| *Serbiona mehadiensis* | KJ912438 | KJ912558 | KJ911984 | MPL337 | KJ912095 | KJ912322 |

* Some sequences from Pérez-Losada et al. (2015) were directly acquired from its senior author and had not become publicly available in GenBank at the time this manuscript was submitted. Therefore the voucher numbers (MPLs) were listed instead.
