# Supplementary material for: Molecular phylogeny and systematics of native North American lumbricid earthworms (Clitellata: Megadrili)
Source: PLoS One. 2017 Aug 9;12(8):e0181504. doi: 10.1371/journal.pone.0181504 (PMC5549934; doi:10.1371/journal.pone.0181504)
Supplement: S2 Table — (DOCX) [file pone.0181504.s002.docx]

S2 Table. Primers used in polymerase chain reactions (PCR).

| Primer pair  (Forward / reverse) | Target gene | Primer sequence  (Forward / reverse) | Reference |
| --- | --- | --- | --- |
| 12SE1 / 12SH | 12S rRNA | 5’-AAA ACA TGG ATT AGA TAC CCR YCT AT-3’ / 5’-ACC TAC TTT GTT ACG ACT TAT CT-5’ | Jamieson et al., 2002 |
| 16Sar / 16Sbr | 16S rRNA | 5’-CGC CTG TTT ATC AAA AAC AT-3’ / 5’-CCG GTC TGA ACT CAG ATC ACG T-3 | Palumbi, 1991 |
| LCO1490 / HCO2198 | COI | 5’-GGT CAA CAA ATC ATA AAG ATA TTG G-3’ / 5’-TAA ACT TCA GGG TGA CCA AAA AAT CA-3’ | Folmer et al., 1994 |
| COIIF1 / COIIR2 | COII | 5’-GGC ACC TAT TTG TTA ATT AGG-3’ / 5’-GTG AGG CAT AGA AAT ACA CC-3’ | Pérez-Losada et al., 2009 |
| ND1F2 / ND1R1b | ND1 | 5’-GAA TAG TGC CAC AGG TTT AAA C-3’ / 5’-TTA ACG TCA TCA GAG TTA TC-3’ | Pérez-Losada et al., 2009 |
| 18F35 / 18R1779 | 18S rRNA | 5’-TCT CAA AGA TTA AGC CAT GCA-3’ / 5’-TGT TAC GAC TTT TAC TTC CTC TA-3’ | Cech et al., 2005 |
| ITS-1 / ITS-2 | 18S-ITS1-5.8S- ITS2-28S | 5’-CGC CCG TCG CTA CTA CCG ATT-3’ / 5’-GTC CCG AAC ACC ACA GTT CCC-3’ | Hong et al., 2007 |
| C1’ / D2 | 28S rRNA | 5’-ACC CGC TGA ATT TAA GCA T-3’ / 5’-TCC GTG TTT CAA GAC GG-3’ | Jamieson et al., 2002 |
